# Serotonin transporter inhibits antitumor immunity through regulating the intratumoral serotonin axis

**DOI:** 10.1016/j.cell.2025.04.032

**Published:** 2025-05-21

**Authors:** Bo Li, James Elsten-Brown, Miao Li, Enbo Zhu, Zhe Li, Yuning Chen, Elliot Kang, Feiyang Ma, Jennifer Chiang, Yan-Ruide Li, Yichen Zhu, Jie Huang, Audrey Fung, Quentin Scarborough, Robin Cadd, Jin J. Zhou, Arnold I. Chin, Matteo Pellegrini, Lili Yang

**Affiliations:** 1 Department of Microbiology, Immunology and Molecular Genetics, University of California, Los Angeles, Los Angeles, CA 90095, USA; 2 Department of Materials Science and Engineering, University of California, Los Angeles, Los Angeles, CA 90095, USA; 3 Department of Molecular, Cell and Developmental Biology, University of California, Los Angeles, Los Angeles, CA 90095, USA; 4 Department of Biostatistics, Fielding School of Public Health, University of California, Los Angeles, Los Angeles, CA, USA; 5 Department of Urology, University of California, Los Angeles, Los Angeles, CA 90095, USA; 6 Eli & Edythe Broad Center of Regenerative Medicine and Stem Cell Research, University of California, Los Angeles, Los Angeles, CA 90095, USA; 7 Jonsson Comprehensive Cancer Center, David Geffen School of Medicine, University of California, Los Angeles, Los Angeles, CA 90095, USA; 8 Bioinformatics Interdepartmental Program, University of California, Los Angeles, Los Angeles, CA 90095, USA; 9 Institute for Quantitative and Computational Biosciences-The Collaboratory, University of California, Los Angeles, Los Angeles, CA 90095, USA; 10 Department of Bioengineering, University of California, Los Angeles, Los Angeles, CA 90095, USA; 11 Goodman-Luskin Microbiome Center, University of California, Los Angeles, Los Angeles, CA 90095, USA; 12 Molecular Biology Institute, University of California, Los Angeles, Los Angeles, CA 90095, USA; 13 Parker Institute for Cancer Immunotherapy, University of California, Los Angeles, Los Angeles, CA 90095, USA; 14 Lead contact

## Abstract

Identifying additional immune checkpoints hindering antitumor T cell responses is key to the development of next-generation cancer immunotherapies. Here, we report the induction of serotonin transporter (SERT), a regulator of serotonin levels and physiological functions in the brain and peripheral tissues, in tumor-infiltrating CD8 T cells. Inhibition of SERT using selective serotonin reuptake inhibitors (SSRIs), the most widely prescribed antidepressants, significantly suppressed tumor growth and enhanced T cell antitumor immunity in various mouse syngeneic and human xenograft tumor models. Importantly, SSRI treatment exhibited significant therapeutic synergy with programmed cell death protein 1 (PD-1) blockade, and clinical data correlation studies negatively associated intratumoral *SERT* expression with patient survival in a range of cancers. Mechanistically, SERT functions as a negative-feedback regulator inhibiting CD8 T cell reactivities by depleting intratumoral T cell-autocrine serotonin. These findings highlight the significance of the intratumoral serotonin axis and identify SERT as an immune checkpoint, positioning SSRIs as promising candidates for cancer immunotherapy.

## In brief

Serotonin transporter (SERT) inhibits CD8 T cell antitumor immunity by depleting intratumoral serotonin. SERT-blocking selective serotonin reuptake inhibitor (SSRI) antidepressants enhance CD8 T cell antitumor immunity and synergize with anti-PD-1, highlighting SSRIs as potential cancer immunotherapeutics.

## Graphical Abstract

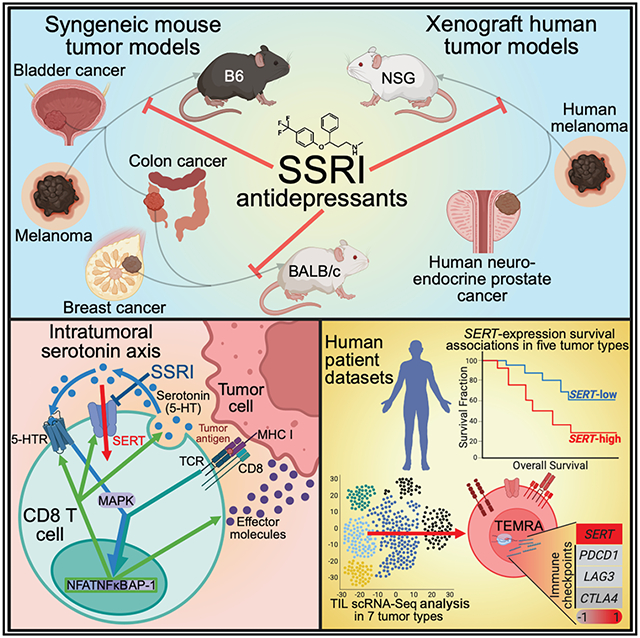

## INTRODUCTION

Immune checkpoint blockade (ICB) is a potent strategy of cancer immunotherapy that combats the immunosuppressive nature of tumors by antagonizing negative immune regulators and enhancing the host antitumor response.^1–4^ As of 2024, 13 ICB antibody therapies had been approved by the U.S. Food and Drug Administration (FDA) for solid tumors, targeting cytotoxic T-lymphocyte antigen 4 (CTLA-4), programmed cell death protein 1 (PD-1), programmed cell death ligand 1 (PD-L1), or lymphocyte activation gene 3 (LAG-3).^5–10^ Despite remarkable cases of complete remission in some patients,^11^ ICB efficacy is limited by individual patient characteristics, as the relevance of specific checkpoints varies across tumor types and distinct tumor microenvironments (TMEs).^5,12–15^ ICB therapies only have an effect in about 15%–25% of treated patients, many of whom suffer tumor relapse.^6,16^ While some progress has been made in combining existing ICB treatments to enhance efficacy,^16,17^ identification of nonredundant immunomodulatory molecules that reshape the TME to better support a potent immune response remains a focus of ongoing cancer immunotherapy research.^18,19^

5-Hydroxytryptamine (5-HT), commonly known as serotonin, is a signaling molecule with diverse functions throughout the body.^20–22^ Although serotonin is widely recognized as a neurotransmitter regulating sleep, mood, and behavior in the central nervous system (CNS), only about 5% of the body’s serotonin is synthesized in the brain,^23,24^ with the vast majority produced in the gut.^25^ Enteric serotonin is derived from two distinct yet highly interconnected sources: neuronal serotonin produced by the enteric nervous system (ENS), which primarily regulates intestinal motility, and mucosal serotonin synthesized by enterochromaffin cells (ECs) in the intestinal lumen, constituting over 90% of the enteric serotonin supply.^26,27^ Beyond its roles in regulating gut motility, inflammation, and communication with the ENS, mucosal serotonin is transported via platelets to peripheral tissues,^26–29^ where serotonin serves as a critical signaling molecule regulating physiological processes, including glucose metabolism, adipogenesis, insulin secretion, and tissue regeneration.^26,27,30–32^

The molecular network regulating serotonin and its biology, or the “serotonin axis,” involves tryptophan hydroxylase 1/2 (TPH1/2) and monoamine oxidase A (MAO-A), enzymes responsible for the synthesis and degradation of serotonin, respectively; the 5-HT receptors (5-HTRs), responsible for detecting serotonin and transmitting signals; and the serotonin transporter (SERT), responsible for regulating serotonin concentrations by transporting serotonin from the extracellular to the intracellular environment.^33,34^ Given the impact of serotonin axis dysregulation in the brain on clinical depression and other psychiatric disorders, various antidepressants have been developed to target these serotonergic proteins.^18^ The most popular class of antidepressants is selective serotonin reuptake inhibitors (SSRIs), which block SERT, increasing extracellular serotonin and promoting 5-HTR activation.^35–38^ These drugs, such as fluoxetine (FLX; trade name Prozac), citalopram (CIT; trade name Celexa), and sertraline (SRT; trade name Zoloft), have gained widespread popularity with the boom in antidepressant use, largely due to their favorable safety profile.^35,36,39^

The molecular machinery of the serotonergic system has long been known to exist in various immune cells, yet the precise role of serotonin in immunoregulation remains underexplored.^19,40–43^ Mucosal serotonin is broadly recognized as proinflammatory in conditions such as colitis,^44–46^ and high peripheral serotonin has been linked to inflammatory diseases, including arthritis and lupus.^42,45,47^ Under inflammatory conditions, serotonin is believed to enhance the activation of dendritic cells (DCs) and macrophages, promoting their antigen-presenting and cytokine-secreting functions.^40,43,48^ Moreover, there is *in vitro* evidence for serotonin’s role as a mitogenic and immunostimulatory signal for effector T cells.^40,43,49,50^ On the other hand, serotonin has been reported to reduce inflammation via 5-HTR4 signaling in the ENS *in vivo* and to promote macrophage alternative activation via 5-HTR7 signaling *in vitro*, suggesting a highly dynamic and tissue context-dependent role of serotonin in immunoregulation.^51–53^ Despite these insights, the role of the peripheral serotonin axis in immune regulation, especially in cancer immunity, remains largely unknown.

Our recent studies explored this field and identified MAO-A as a potent regulator of immune activity within the TME.^19,54^ MAO-A inhibitors (MAOIs) were shown to enhance CD8 T cell reactivities and depolarize immunosuppressive tumor-associated macrophages (TAMs).^18,19,54^ This implication of an immunomodulatory serotonin axis in the tumor turned our focus to SERT, a potent regulator and primary drug target of the serotonin axis in the CNS.^33,34^ In this study, we investigated the role of SERT in regulating the intratumoral serotonin axis and CD8 T cell responses against solid tumors and evaluated the potential clinical relevance of SSRIs in co-opting this pathway to enhance cancer immunotherapy using genetic and pharmacological approaches, preclinical syngeneic and human xenograft mouse tumor models, and clinical correlative analyses of a wide array of solid tumor patients.

## RESULTS

### SERT blockade suppresses tumor growth and enhances cytotoxic CD8 T cell antitumor responses in multiple syngeneic mouse tumor models

**Figure 1.**
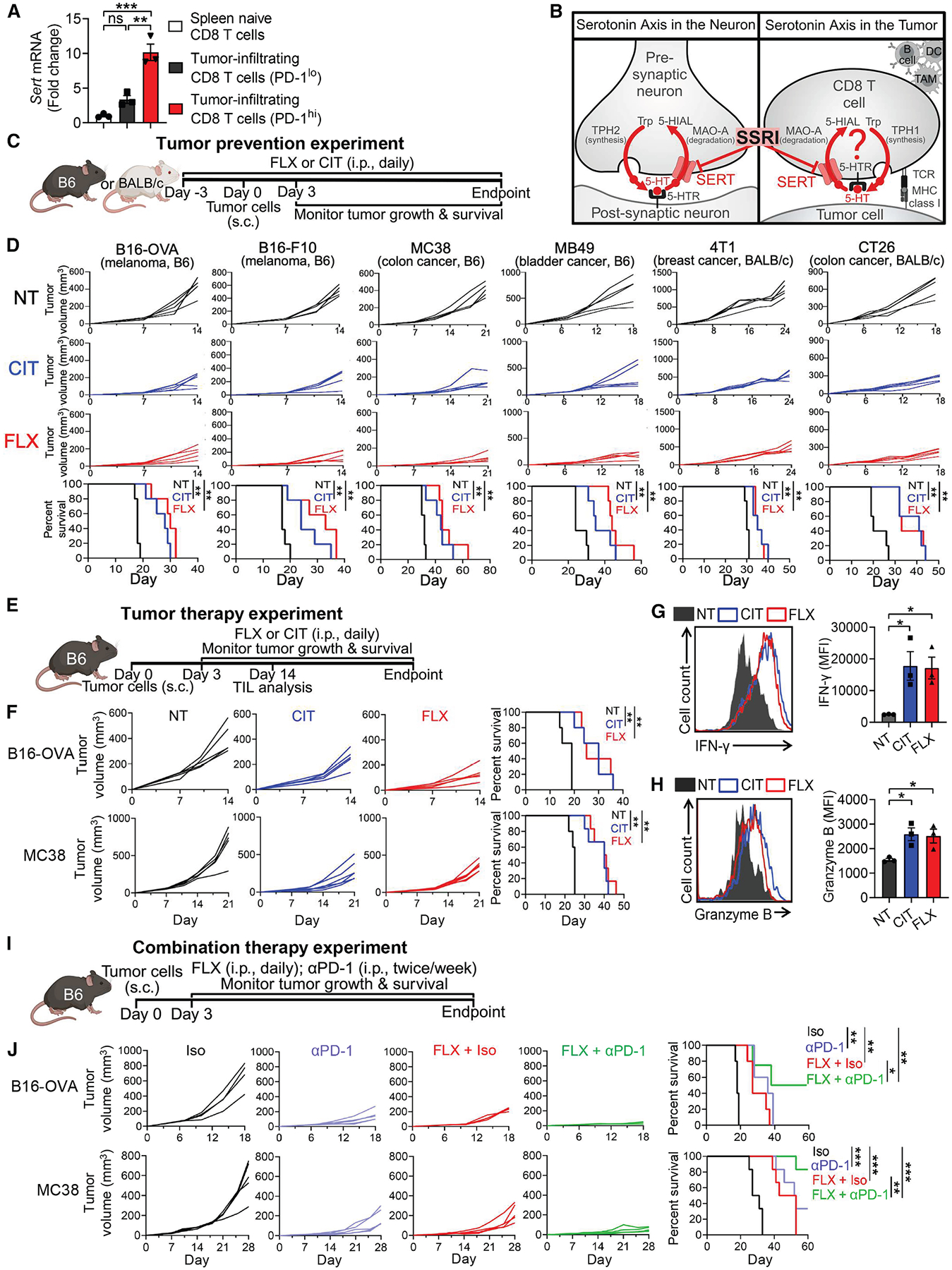
SERT blockade suppresses tumor growth and enhances cytotoxic CD8 T cell antitumor responses in multiple syngeneic mouse tumor models (A) Quantitative reverse-transcription PCR (RT-qPCR) analyses of *Sert* mRNA expression in tumor-infiltrating CD8 T cell subsets (gated as CD45.2^+^TCRβ^+^CD8^+^PD-1^lo^ or CD45.2^+^TCRβ^+^CD8^+^PD-1^hi^) isolated from day 14 B16-OVA tumors grown in WT B6 mice (*n* = 3). Naive CD8 T cells (gated as TCRβ^+^CD8^+^CD44^lo^CD62L^hi^) sorted from the spleen of tumor-free B6 mice were included as a control (*n* = 3). (B) Schematics showing the intratumoral serotonin axis in analogy to the serotonin axis in the neuron. In an antitumor CD8 T cell, TPH1 converts Trp into 5-HT, which is secreted into the tumor microenvironment (TME) and in turn stimulates the T cell by binding to its surface 5-HTR. SERT depletes 5-HT from the TME by transporting it back into the T cell, where it is degraded by MAO-A into 5-HIAL. SSRIs can inhibit SERT activity, increasing extracellular 5-HT and preventing degradation. TPH1/2, tryptophan hydroxylase 1/2; Trp, tryptophan; 5-HT, 5-hydroxytryptamine or serotonin; 5-HTR, 5-HT receptor; SERT, serotonin transporter; MAO-A, monoamine oxidase A; 5-HIAL, 5-hydroxyindolealdehyde; SSRIs, selective serotonin reuptake inhibitors; TCR, T cell receptor; MHC, major histocompatibility complex; DC, dendritic cell; TAM, tumor-associated macrophage. (C and D) SSRI treatment in tumor prevention experiments. (C) Experimental design. Six syngeneic mouse tumor models and two SSRIs, fluoxetine (FLX; trade name Prozac) and citalopram (CIT; trade name Celexa), were used. s.c., subcutaneous. (D) Tumor growth and animal survival (*n* = 5). NT, non-treated. (E–H) SSRI treatment in tumor therapy experiments. (E) Experimental design. Two syngeneic mouse tumor models (B16-OVA and MC38) and two SSRIs (FLX and CIT) were used. (F) Tumor growth and animal survival (*n* = 5). (G and H) Fluorescence-activated cell-sorting (FACS) analysis of intracellular IFN-γ (G) and Granzyme B (H) production in tumor-infiltrating CD8 T cells isolated from B16-OVA tumors at day 14 (*n* = 3). MFI, median fluorescence intensity. (I and J) SSRI and anti-PD-1 combination treatment in tumor therapy experiments. (I) Experimental design. Two syngeneic mouse tumor models (B16-OVA and MC38) and one SSRI (FLX) were used. (J) Tumor growth and animal survival (*n* = 5). Iso, isotype control; αPD-1, anti-PD-1. Representative of two (A, D, G, and H) and three (F and J) experiments. Data are presented as the mean ± SEM. ns, not significant, *p < 0.05, **p < 0.01, and ***p < 0.001 by one-way ANOVA (A, G, and H) or log rank (Mantel-Cox) test adjusted for multiple comparisons (D, F, and J). See also Figures S1 and S7.

To investigate the possible involvement of SERT in CD8 T cell antitumor responses, we first isolated tumor-infiltrating CD8 T cells from a B16-ovalbumin (B16-OVA) mouse melanoma model and examined *Sert* gene expression. We detected an overall upregulation of *Sert* gene expression in tumor-infiltrating CD8 T cells compared with their naive counterparts, with PD-1^hi^ cells showing higher induction of *Sert* expression than PD-1^lo^ cells (Figure 1A). This correlation suggests that SERT may regulate the antitumor responses and exhaustion status of CD8 T cells. We then hypothesized that SERT blockade with established SSRIs might impact the antitumor immunity, particularly the CD8 T cell antitumor responses, via modulating the intratumoral serotonin axis (Figure 1B).

To represent SSRIs, we used FLX and CIT, two of the most-prescribed drugs of their class.^35,36^ The SSRI doses used in our animal studies reflect therapeutical doses in human, producing comparable serum SSRI levels.^55–59^ Safety of the SSRI treatments in our animal studies was validated by a lack of exaggerated tissue inflammation (Figure S1A), autoantibody induction (Figure S1B), and systemic peripheral T cell proliferation and hyperactivation outside of tumors (Figures S1C–S1E).

Six syngeneic mouse models across two mouse strains (C57BL/6J or B6 and BALB/c) were used in our study, spanning melanoma (B16-F10) and ovalbumin-expressing melanoma (B16-OVA), colon cancer (MC38 and CT26), bladder cancer (MB49), and breast cancer (4T1). In a set of tumor prevention experiments, administration of FLX or CIT resulted in greatly reduced tumor growth and prolonged animal survival in all six models regardless of tumor type or mouse genetic background (Figures 1C and 1D).

Encouraged by the tumor prevention efficacy of SSRIs, we next evaluated the therapeutic potential of SSRIs in tumor therapy experiments employing the B16-OVA melanoma and MC38 colon cancer models (Figure 1E). SSRI treatment significantly reduced tumor growth and improved animal survival in both models (Figure 1F). Analysis of day 14 B16-OVA tumors revealed increased CD8 T cell abundance in SSRI-treated tumors (Figure S1F), accompanied by enhanced effector functions, as evidenced by increased intracellular production of interferon (IFN)-γ (Figure 1G) and Granzyme B (Figure 1H). Notably, SSRI treatment led to a significant increase in the TCF1^+^ memory CD8 T cell subset (Figure S1G), indicating a potential role of SERT in regulating the generation and/or maintenance of the “stem-like” memory antitumor CD8 T cells.^60^ Further analysis revealed comparable levels of tumor antigen-specific CD8 T cells in SSRI-treated and control tumors (Figure S1H); however, those in SSRI-treated tumors exhibited an enhanced effector phenotype (Figure S1I).

To better model clinical application, we evaluated the therapeutic potential of SSRIs using a 4T1 mouse breast cancer orthotopic model (Figure S1J), which closely recapitulates the breast tumor microenvironment.^61^ In this model, SSRI treatment effectively suppressed tumor growth (Figure S1K) and enhanced the effector function of intratumoral CD8 T cells, as evidenced by their increased production of the key cytotoxic molecule Granzyme B (Figure S1L), further supporting the cancer therapy potential of SSRIs.

Next, we explored the potential of SSRIs for combination therapy, combining FLX and anti-PD-1 treatments (Figure 1I). In both B16-OVA melanoma and MC38 colon cancer models, FLX and anti-PD-1 monotherapies similarly impeded tumor growth (Figure 1J). Combination therapy in the B16-OVA model, which is relatively insensitive to traditional ICB therapies,^62^ yielded impressive synergistic efficacy (Figure 1J). Although anti-PD-1 monotherapy resulted in robust antitumor efficacy in the MC38 tumor model, which is sensitive to anti-PD-1,^63^ combination therapy further improved the antitumor effect of anti-PD-1 treatment (Figure 1J).

Collectively, these findings demonstrate the immunotherapeutic potential of SSRIs in a broad range of cancers and suggest that SERT regulates CD8 T cell antitumor immunity through a possible intratumoral serotonin axis (Figure 1B).

### SERT blockade enhances antitumor CD8 T cell effector and proliferating gene profiles

**Figure 2.**
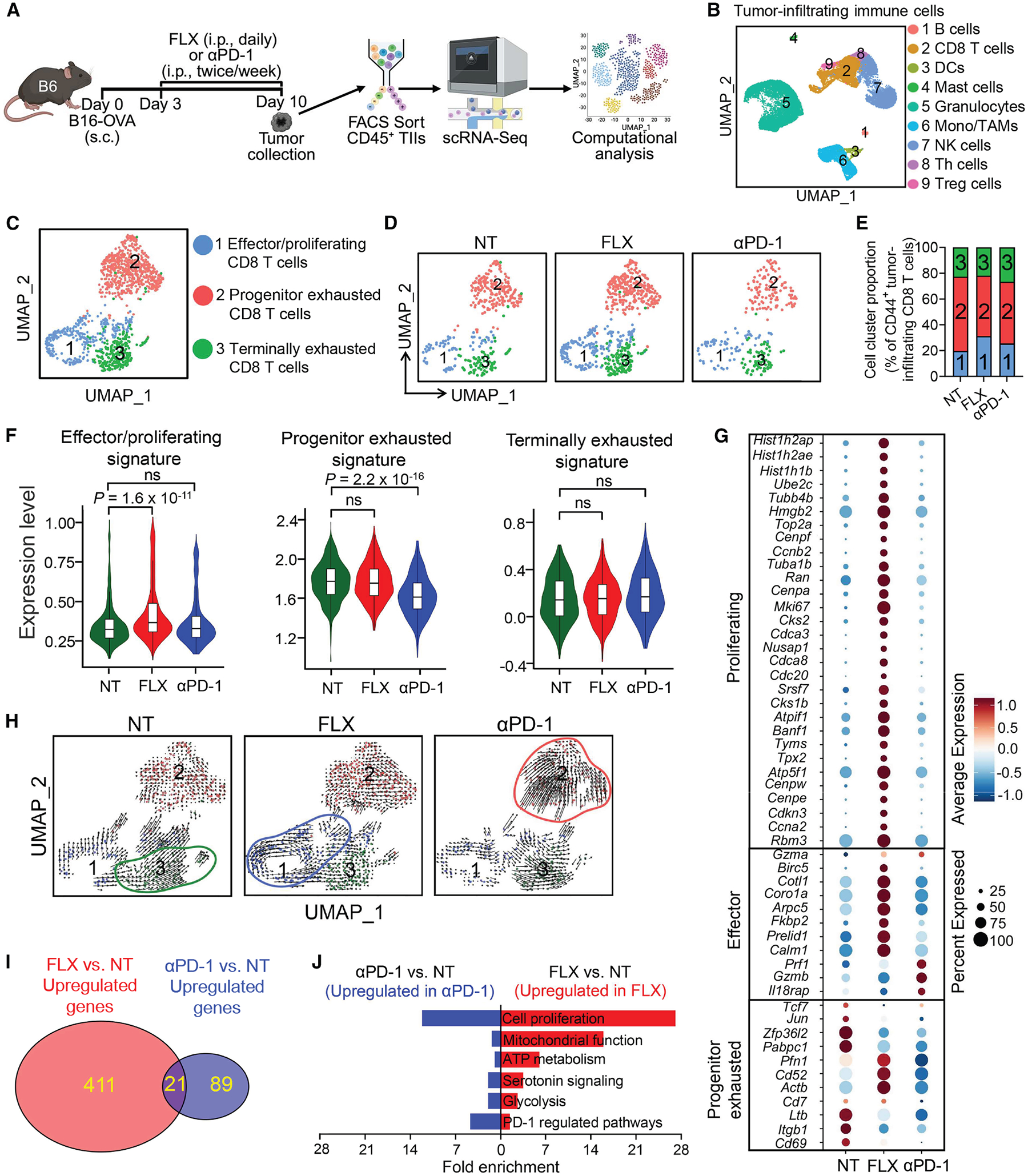
SERT blockade enhances antitumor CD8 T cell effector and proliferating gene profiles (A) Schematics showing the experimental design to study the *in vivo* gene profiling of antitumor CD8 T cells using scRNA-seq. CD45^+^ tumor-infiltrating immune cells were sorted from day 10 B16-OVA tumors and then subjected to scRNA-seq analysis. Three experimental groups were included: non-treated (NT), FLX-treated (FLX), and anti-PD-1-treated (αPD-1). (B) scRNA-seq analysis of the total CD45^+^ tumor-infiltrating immune cells combined from all samples. Combined uniform manifold approximation and projection (UMAP) plot is presented, showing the formation of nine major cell clusters. Each dot represents a single cell and is colored according to its cell cluster assignment. Mono, monocytes; NK, natural killer; Th, T helper; Treg, CD4 regulatory T. (C–J) scRNA-seq analysis of the antigen-experienced (CD44^+^) tumor-infiltrating CD8 T cells identified from (B). (C) Combined UMAP plot showing the formation of three major cell clusters. Each dot represents a single cell and is colored according to its cell cluster assignment. Gene signature profiling analysis identified cluster 1 to be the effector/proliferating CD8 T cells, cluster 2 to be the progenitor exhausted CD8 T cells, and cluster 3 to be the terminally exhausted CD8 T cells. (D) Individual UMAP plots showing the three-cell cluster composition of the indicated treatment groups. (E) Bar graphs showing the cell cluster proportions from (D). (F) Violin plots showing the expression distribution of the indicated gene signatures in each treatment group. Box and whisker plots exhibit the minimum, lower quartile, median, upper quartile, and maximum expression levels of each group. (G) Dot plots showing the expression of representative signature genes in each treatment group. Color saturation indicates the strength of averaged gene expression. The dot size indicates the percentage of cells expressing the indicated genes. (H) RNA velocity projected on UMAP plots. Arrows represent the estimates of local average velocity, showing the path (indicated by arrow orientation) and pace (indicated by arrow length) of cell transition. (I) Venn diagram showing numbers of genes upregulated by FLX or anti-PD-1 treatment. (J) Bar plots showing the fold enrichment of indicated pathways upregulated by FLX or anti-PD-1 treatment. The experiment was performed once, and cells isolated from 10 mice of each experimental group were combined for analysis. The *p* values of violin plots were determined by the Kruskal-Wallis test for the overall comparison and Dunn’s test for post hoc pairwise comparisons between groups (F). *p* < 0.05 was considered significant. ns, not significant. See also Figures S2 and S7.

To investigate how SERT blockade alters tumor-infiltrating CD8 T cell compartment, we isolated CD45^+^ tumor-infiltrating immune cells from B6 mice bearing B16-OVA tumors treated with or without FLX and conducted a single-cell RNA sequencing (scRNA-seq) study (Figure 2A). Tumor-infiltrating immune cells from B6 mice treated with anti-PD-1 were included to compare the effects of SSRI to those of a traditional checkpoint inhibitor (Figure 2A). Uniform manifold approximation and projection (UMAP) analysis of total combined tumor-infiltrating immune cells showed the formation of nine-cell clusters: B cells, CD8 T cells, DCs, mast cells, granulocytes, monocytes/TAMs, natural killer (NK) cells, T helper (Th) cells, and CD4 regulatory T (Treg) cells (Figures 2B and S2A). Cell cluster distributions were similar between non-treated and FLX-treated tumor-infiltrating immune cells, and notably, anti-PD-1-treated cells comprised a significantly higher proportion of granulocytes (Figures S2B and S2C), in agreement with previous reports.^64–66^

Further UMAP analysis of the antigen-experienced cytotoxic CD8 T cell (CD8^+^CD44^+^) cluster revealed three major subclusters (Figure 2C). These consisted of effector/proliferating (cluster 1), progenitor exhausted (cluster 2), and terminally exhausted (cluster 3) CD8 T cells (Figure 2C), as identified by gene signature analysis (Figures S2D, S2F, and S2G) and gene set enrichment analysis (GSEA; Figure S2E). Compared with the non-treated control, the FLX-treated tumor-infiltrating CD8 T cells exhibited an enrichment in effector/proliferating (cluster 1) cells, accompanied by a reduction in exhausted (clusters 2 and 3) cells (Figures 2D and 2E).

Consistently, gene signature analysis showed an overall enhancement of the T cell effector/proliferating signature in CD8 T cells from FLX-treated mice (Figures 2F and 2G). FLX treatment also upregulated genes associated with mitochondrial function, which play a key role in CD8 T cell activation and persistence (i.e., mitochondrial electron transport chain genes; Figures S2F, S2H, and S2I). On the other hand, the anti-PD-1 treatment primarily decreased the proportion of the progenitor exhausted (cluster 2) cells (Figures 2D and 2E) and greatly reduced the overall expression of progenitor exhausted CD8 T cell signature genes (Figures 2F and 2G). These interesting findings were further validated by RNA-velocity analysis showing that SSRI and anti-PD-1 treatment resulted in a relative accumulation of tumor-infiltrating CD8 T cells at the effector/proliferating stage (cluster 1) and the progenitor exhausted stage (cluster 2), respectively (Figure 2H).

Venn diagram analysis revealed that there was very little overlap between the total upregulated genes in FLX-treated and anti-PD-1-treated tumor-infiltrating CD8 T cells (21/432 and 21/110, respectively; Figure 2I). Notably, the FLX treatment-induced gene set (432 genes) was significantly larger than that induced by the anti-PD-1 treatment (110 genes; Figure 2I). We then performed pathway analysis for those genes specifically upregulated by FLX treatment. FLX treatment-enriched pathways were related to cell proliferation, mitochondrial function, ATP metabolism, serotonin signaling, and glycolysis, and these pathways had a substantially lower enrichment in anti-PD-1-treated CD8 T cells (Figure 2J), suggesting SERT may regulate reactivities of tumor-infiltrating CD8 T cells via mechanisms distinct to those associated with PD-1.

Together, these *in vivo* gene profiling studies suggest SERT may play an important role in regulating the generation/persistence of antitumor effector CD8 T cells.

### SERT functions as a T cell-intrinsic factor negatively regulating CD8 T cell-mediated antitumor responses

**Figure 3.**
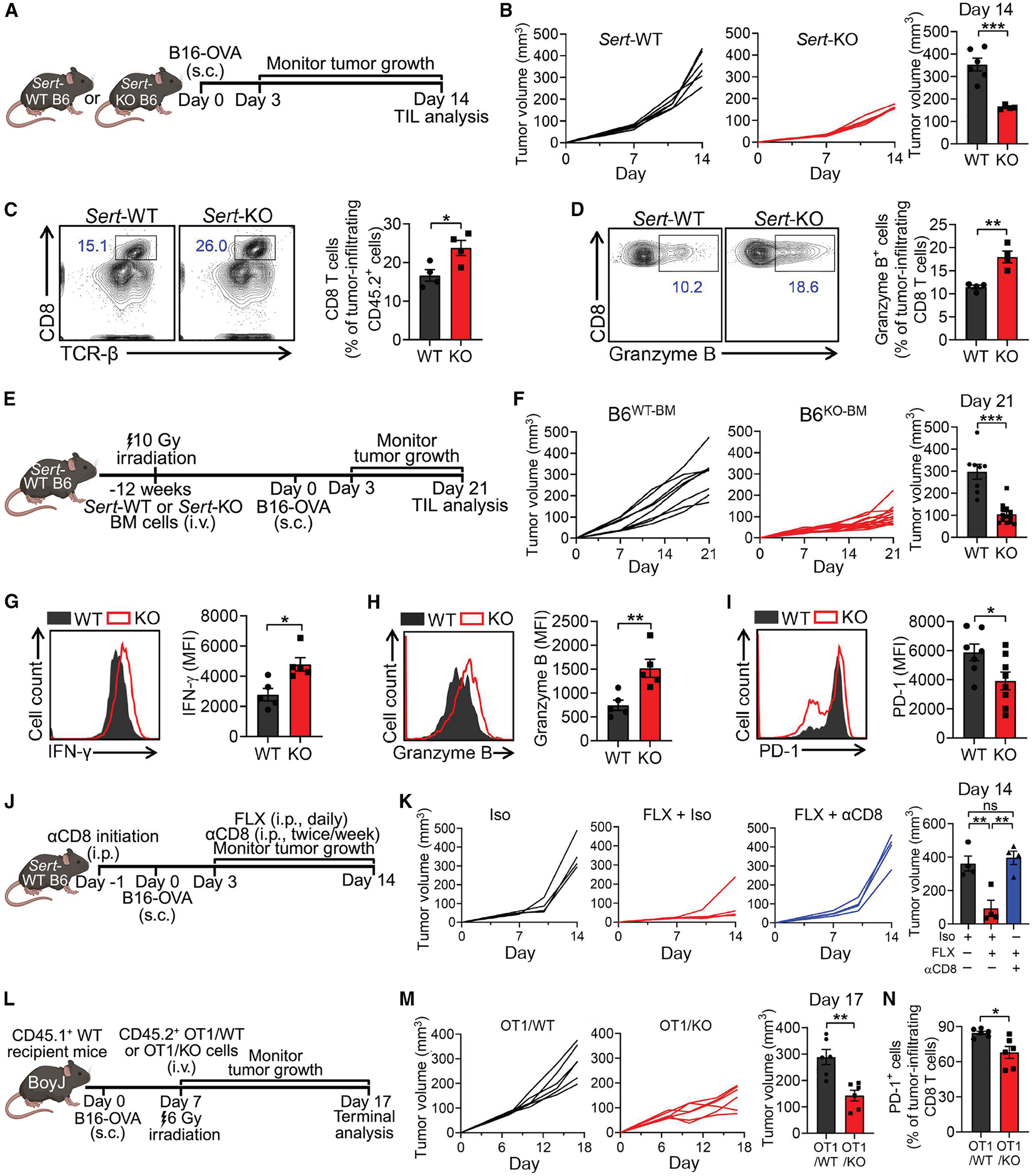
SERT functions as a T cell-intrinsic factor negatively regulating CD8 T cell-mediated antitumor responses (A–D) *Sert*-WT and *Sert*-KO mice tumor challenge experiments. (A) Experimental design. (B) Tumor growth (*n* = 4–6). (C and D) FACS analyses of the numbers(C) and intracellular Granzyme B production (D) of tumor-infiltrating CD8 T cells at day 14 (*n* = 4). (E–I) Bone marrow (BM) transfer experiments. (E) Experimental design. (F) Tumor growth (*n* = 8–13). (G and H) FACS analyses of intracellular IFN-γ (G) and Granzyme B (H) production in tumor-infiltrating CD8 T cells isolated from day 21 tumors (*n* = 5). (I) FACS analyses of PD-1 expression on tumor-infiltrating CD8 T cells isolated from day 21 tumors (*n* = 7–8). (J and K) CD8 T cell depletion experiments. (J) Experimental design. (K) Tumor growth (*n* = 4). αCD8, anti-CD8. (L–N) OT1 T cell adoptive transfer experiment. (L) Experimental design. i.v., intravenous. (M) Tumor growth (*n* = 6). (N) FACS analyses of surface PD-1 expression on tumor-infiltrating CD8 T cells isolated from day 17 B16-OVA tumors (*n* = 6). Representative of one (M and N), two (B–D and F–I), and three (K) experiments. Data are presented as the mean ± SEM. ns, not significant, *p < 0.05, **p < 0.01, and ***p < 0.001 by Student’s t test (B–D, F–I, M, and N) or one-way ANOVA (K). See also Figure S3.

To further study the role of SERT in regulating CD8 T cell anti-tumor responses, we utilized the *Sert* knockout (*Sert*-KO) mice and included the wild-type (WT) B6 mice as the *Sert*-WT control (Figure 3A). *Sert*-KO mice carry a *Sert* mutant with targeted deletion of exon 2 that results in expression of nonfunctional SERT protein.^67^ Before B16-OVA melanoma challenge, these mice exhibited normal numbers of T cells in the periphery (Figure S3A), which displayed a typical naive phenotype (CD25^lo^CD44^lo^CD62L^hi^; Figure S3B). After tumor challenge, *Sert*-KO mice displayed significantly suppressed tumor growth (Figure 3B) and possessed increased numbers of tumor-infiltrating CD8 T cells (Figure 3C) exhibiting enhanced effector function (i.e., increased Granzyme B production; Figure 3D).

Next, we performed a bone marrow transfer (BMT) experiment that confined SERT deficiency to immune cells by reconstituting B6 recipient mice with bone marrow (BM) cells from either *Sert*-WT or *Sert*-KO donor mice, followed by B16-OVA tumor challenge (Figure 3E). Consistent with global *Sert*-KO mice, *Sert*-KO BMT mice showed a dramatically delayed B16-OVA tumor growth (Figure 3F). Tumor-infiltrating CD8 T cells from *Sert*-KO BMT mice displayed enhanced effector function (i.e., higher IFN-γ and Granzyme B production; Figures 3G and 3H) and decreased exhaustion markers (i.e., PD-1; Figure 3I). Therefore, SERT can function as an immune cell-intrinsic regulator for anti-tumor responses.

To further assess the importance of CD8 T cells in SERT-mediated tumor control, we depleted CD8 T cells in *Sert*-WT mice challenged with B16-OVA melanoma, followed by FLX treatment (Figure 3J). CD8 T cell depletion abolished the FLX-induced tumor suppression effect (Figure 3K). We also grew B16-OVA tumors in immunodeficient NOD *scid* gamma (NSG) mice, which lack mature lymphocytes (Figure S3C). Treatment with SSRI (FLX or CIT) did not suppress the *in vivo* growth of B16-OVA tumors in NSG mice (Figure S3D).

To investigate whether SERT directly regulates the antitumor activity of CD8 T cells, we bred *Sert*-KO mice with OT1 transgenic (*OT1*-Tg) mice and generated *OT1*-Tg/*Sert*-KO mice producing OVA-specific CD8 T cells deficient in SERT (Figure S3E). OT1 T cells were isolated from either *OT1*-Tg or *OT1*-Tg/*Sert*-KO mice (denoted as OT1/WT or OT1/KO T cells, respectively) and transferred separately into CD45.1 WT mice bearing pre-established B16-OVA tumors (Figures 3L and S3F). This experimental design restricted the comparison of SERT deficiency to tumor antigen-specific OT1 T cells. Both OT1/WT and OT1/KO T cells effectively infiltrated the tumors; however, OT1/KO T cells exhibited significantly enhanced tumor control (Figure 3M), which was associated with enhanced effector function (Figure S3G) and a reduced exhaustion phenotype (Figures 3N, S3H, and S3I).

Collectively, these *in vivo* findings identify SERT as a T cell-intrinsic factor negatively regulating CD8 T cell antitumor immunity.

### SERT acts as an autonomous factor negatively regulating CD8 T cell antigen responses

**Figure 4.**
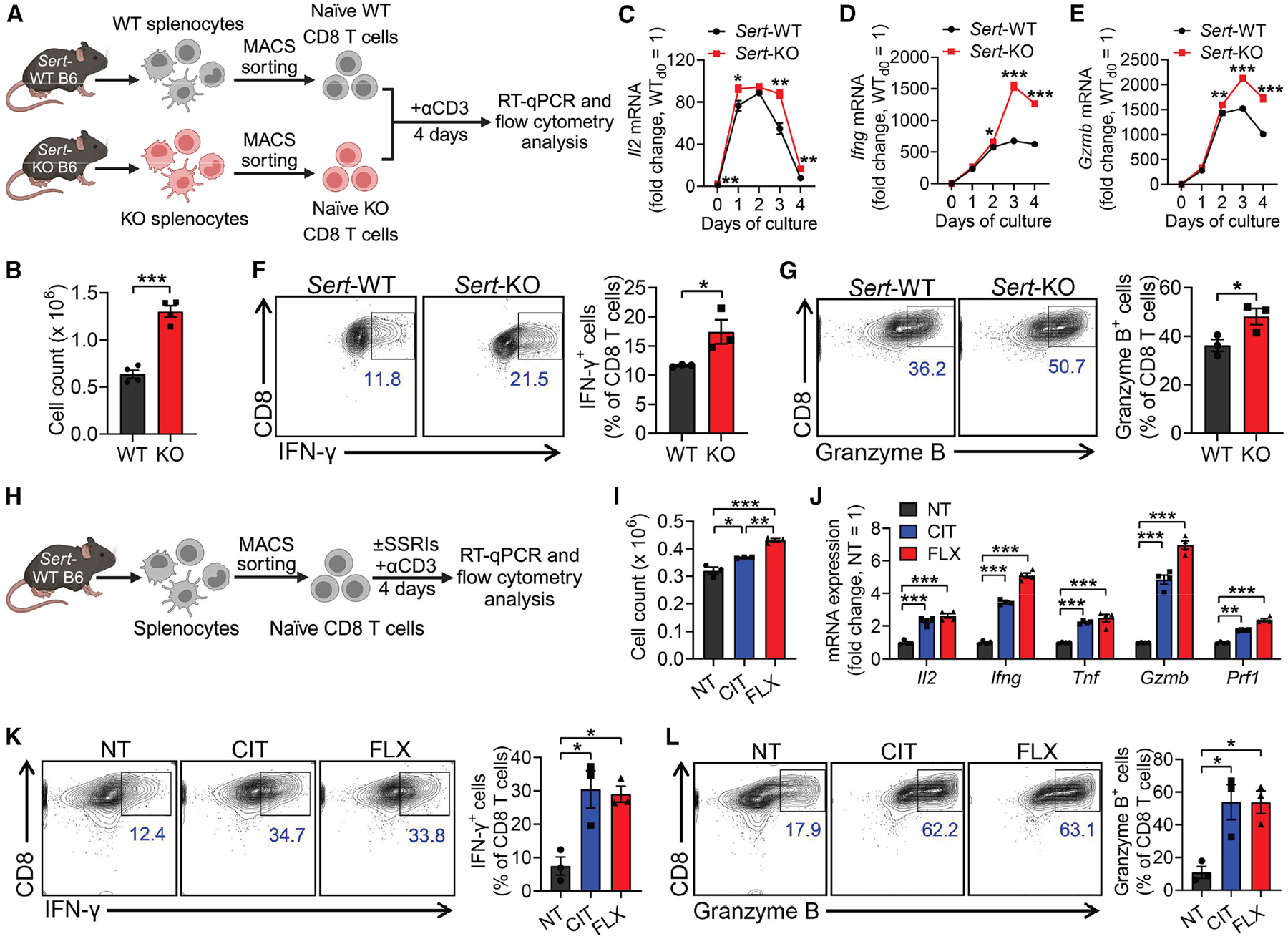
SERT acts as an autonomous factor negatively regulating CD8 T cell antigen responses (A–G) CD8 T cell antigen response in the absence of SERT. (A) Experimental design. Naive CD8 T cells were purified from *Sert*-WT and *Sert*-KO B6 mice and stimulated *in vitro* with anti-CD3 over 4 days. (B) Cell counts at day 4 (*n* = 4). (C–E) RT-qPCR analyses of *Il2* (C), *Ifng* (D), and *Gzmb* (E) expression over time (*n* = 4). (F and G) FACS analyses of intracellular IFN-γ (F) and Granzyme B (G) production at day 3 (*n* = 3). αCD3, anti-CD3. (H–L) CD8 T cell antigen response under SSRI treatment. (H) Experimental design. Naive CD8 T cells were purified from *Sert*-WT B6 mice and stimulated *in vitro* with anti-CD3 over 4 days in the presence or absence of SSRI (CIT or FLX) treatment. NT, non-treated. (I) Cell counts at day 3 (*n* = 4). (J) RT-qPCR analyses of effector gene (i.e., *Il2*, *Ifng*, *Tnf*, *Gzmb*, *Prf1*) expression at day 2 (*n* = 4). (K and L) FACS analyses of intracellular IFN-γ (K) and Granzyme B (L) production at day 3 (*n* = 3). Representative of two (B–G and I) and three (J–L) experiments. Data are presented as the mean ± SEM. *p < 0.05, **p < 0.01, and ***p < 0.001 by Student’s t test (B–G) or one-way ANOVA (I–L). See also Figure S4.

To assess if SERT acts as an autonomous factor directly regulating CD8 T cell antigen responses, we isolated CD8 T cells from *Sert*-WT or *Sert*-KO mice and stimulated these T cells with anti-CD3 (Figure 4A). *Sert*-KO CD8 T cells showed an enhancement in cell proliferation (Figure 4B), effector cytokine expression (i.e., interleukin [IL]-2 and IFN-γ; Figures 4C, 4D, and 4F), and cytotoxic molecule expression (i.e., Granzyme B; Figures 4E and 4G). Study of *Sert*-KO OVA-specific OT1 T cells gave similar results, suggesting a general role of SERT in regulating CD8 T cells across various antigen specificities (Figures S4A–S4D). Consistent results were also obtained when we stimulated *Sert*-KO CD8 T cells with anti-CD3 and anti-CD28 antibodies (Figures S4E–S4G).

To test if pharmacological inhibition of SERT in *Sert*-WT CD8 T cells can recapitulate the *Sert*-KO phenotype, we isolated CD8 T cells from *Sert*-WT mice and stimulated them with anti-CD3 in the presence or absence of an SSRI (FLX or CIT; Figure 4H). SSRI treatments resulted in a significant enhancement in CD8 T cell proliferation (Figure 4I) and expression of effector cytokines (i.e., IL-2, IFN-γ, and tumor necrosis factor alpha [TNF-α]; Figures 4J and 4K) and cytotoxic molecules (i.e., Granzyme B and Perforin; Figures 4J and 4L). This enhancement in CD8 T cell effector function induced by SSRIs was dose-dependent (Figures S4H–S4M). Study of SSRI-treated *Sert*-WT CD8 T cells stimulated with anti-CD3 and anti-CD28 antibodies also yielded similar results (Figures S4N–S4Q).

These *in vitro* studies validate SERT as an autonomous factor restraining CD8 T cell antigen responses in the presence or absence of co-stimulation.

### SERT restrains CD8 T cell antigen responses by directly regulating the autocrine serotonin signaling pathway

**Figure 5.**
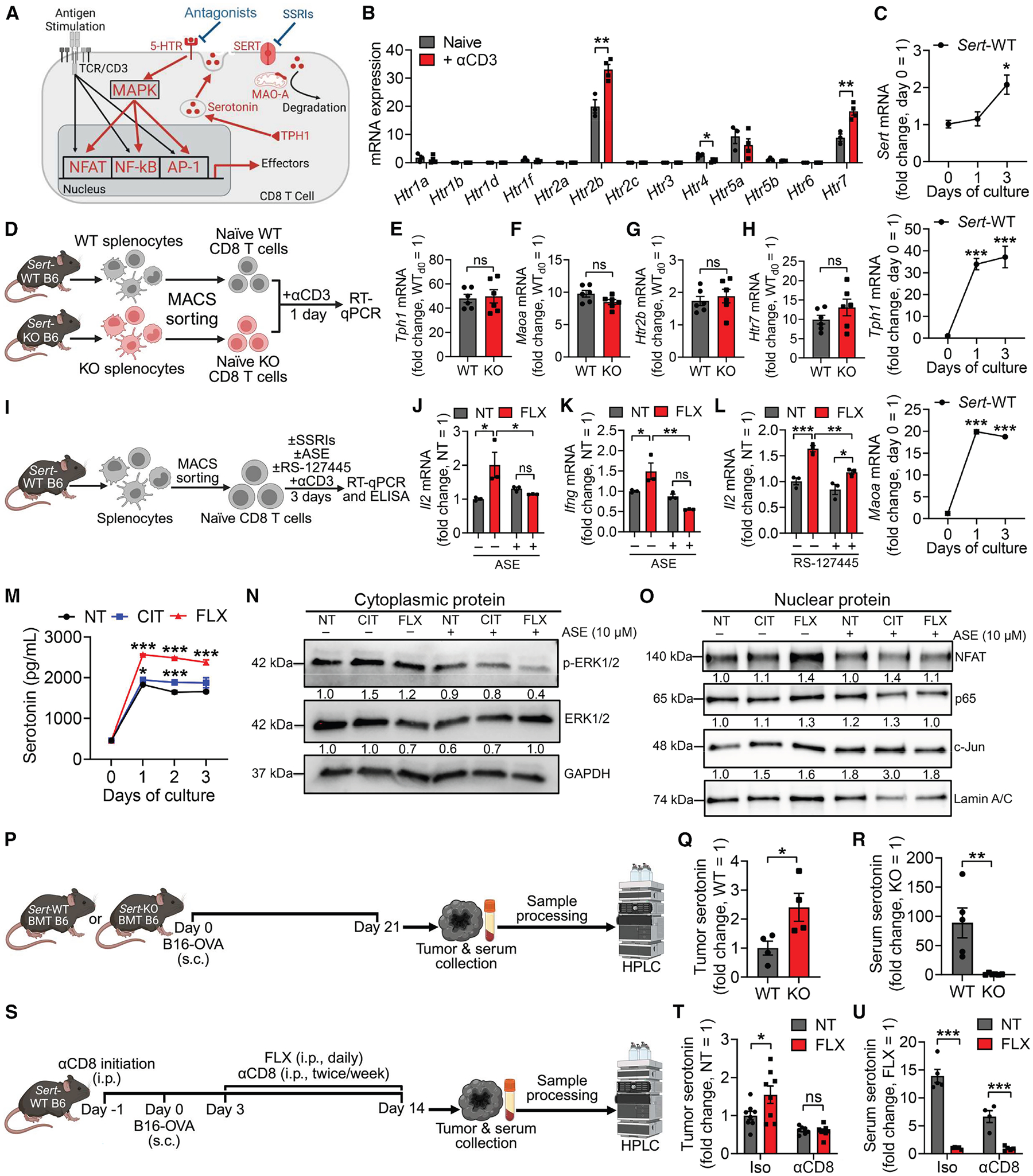
SERT restrains CD8 T cell antigen responses by directly regulating the autocrine serotonin signaling pathway (A) Schematics showing the proposed autocrine serotonin signaling pathway in a CD8 T cell. Possible pharmacological interventions are indicated. (B and C) Serotonergic gene expression in *Sert*-WT CD8 T cells in response to antigen stimulation. Naive CD8 T cells were purified from *Sert*-WT B6 mice and stimulated with anti-CD3 for 3 days. (B) RT-qPCR analyses of 5-HTR family member gene expression at day 1 (*n* = 4). Unstimulated naive CD8 T cells were included as a control. (C) RT-qPCR analyses of *Sert*, *Tph1*, and *Maoa* gene expression over time (*n* = 4). (D–H) Serotonergic gene expression in activated *Sert*-WT and *Sert*-KO CD8 T cells. (D) Experimental design. Naive CD8 T cells were purified from *Sert*-WT and *Sert*-KO B6 mice and stimulated *in vitro* with anti-CD3 for 1 day. (E–H) RT-qPCR analyses of *Tph1* (E), *Maoa* (F), *Htr2b* (G), and *Htr7* (H) expression (*n* = 4). (I–M) Autocrine serotonin signaling in *Sert*-WT CD8 T cells. (I) Experimental design. *Sert*-WT CD8 T cells were stimulated with anti-CD3 for 3 days in serotonin-depleted medium in the presence or absence of SSRI (FLX or CIT) and/or 5-HTR antagonist (ASE or RS-127445) treatment. ASE, asenapine (a general antagonist of most 5-HTR subtypes); RS-127445, a 5-HTR2B selective antagonist. (J and K) RT-qPCR analyses of *Il2* (J) and *Ifng* (K) expression in FLX-treated or non-treated (NT) CD8 T cells at day 2, with or without ASE treatment (*n* = 3). (L) RT-qPCR analyses of *Il2* expression in FLX-treated or non-treated (NT) CD8 T cells at day 2, with or without RS-127445 treatment (*n* = 3). (M) ELISA analyses of serotonin levels over time in culture supernatants of FLX-treated, CIT-treated, or non-treated (NT) CD8 T cells (*n* = 4). (N and O) Western blot analyses of key signaling molecules involved in the 5-HTR-MAPK (N) and TCR (O) signaling pathways. MAPK, mitogen-activated protein kinase. (P–R) Serotonin levels in *Sert*-WT and *Sert*-KO BMT mice bearing B16-OVA tumors (denoted as WT and KO, respectively). (P) Experimental design. (Q and R) HPLC analyses of serotonin levels in tumor (Q, *n* = 4) and serum (R, *n* = 5–6) at day 21. (S–U) Serotonin levels in *Sert*-WT mice bearing B16-OVA tumors, with or without SSRI (i.e., FLX) and/or anti-CD8 depletion antibody treatment. (S) Experimental design. (T and U) HPLC analyses of serotonin levels in tumor (T, *n* = 7–8) and serum (U, *n* = 4–5) at day 14. Representative of two (B, E–H, J–M, N, and O) and three (C, Q, R, T, and U) experiments. Data are presented as the mean ± SEM. ns, not significant, *p < 0.05, **p < 0.01, and ***p < 0.001 by Student’s t test (B, E–H, Q, and R), one-way ANOVA (C and M), or two-way ANOVA with Turkey’s multiple comparisons test (J–L, T, and U). See also Figure S5.

SERT regulates brain activity through modulating the local serotonin axis (Figure 1B), where neurons synthesize and utilize serotonin for synaptic signal transmission.^68,69^ Interestingly, mouse T cells have the same molecular machinery for serotonin regulation (production, transportation, degradation, and reception), and stimulation of surface 5-HTRs has been linked to enhanced T activation and immune function.^19,40^ We therefore proposed a SERT-regulated intratumoral serotonin axis, wherein autocrine serotonin binds to surface 5-HTRs and enhances immune pathways for anti-tumor CD8 T cell activation and effector function (Figure 5A).^19,40^

To test this hypothesis, we first characterized the expression of serotonergic molecular machinery in mouse CD8 T cells. We isolated naive CD8 T cells from B6 WT mice and stimulated these cells *in vitro* with anti-CD3 to mimic antigen stimulation. In a panel of all 13 mouse 5-HTR subtypes, only two subtype genes (*Htr2b* and *Htr7*) were expressed at high levels, both of which were further induced by antigen stimulation (Figure 5B). Interestingly, *Htr7*-KO CD8 T cells exhibited markedly reduced effector function after antigen stimulation (Figures S5A–S5G). In *Sert*-WT CD8 T cells, antigen stimulation significantly upregulated the expression of *Sert*, *Tph1*, and *Maoa* genes, which encode the key proteins regulating serotonin transport, synthesis, and degradation, respectively (Figures 5C and S5H).^18^ Consistently, these key serotonergic genes were dramatically upregulated in antigen-experienced (PD-1^hi^) tumor-infiltrating CD8 T cells *in vivo* (Figures 1A and S5I–S5L). These findings indicate an antigen-induced upregulation of the serotonergic system in mouse CD8 T cells and suggest SERT may act as a negative-feedback regulator inhibiting CD8 T cell activities.

Next, we sought to determine whether SERT deficiency influenced the expression of other serotonergic molecules. Comparison of serotonergic gene expression between antigen-stimulated *Sert*-WT and *Sert*-KO CD8 T cells (Figure 5D) showed comparable levels of *Tph1*, *Maoa*, *Htr2b*, and *Htr7* genes (Figures 5E–5H), indicating that SERT regulation of CD8 T cell activation is not achieved through modulating other key serotonergic molecules.

To examine the role of autocrine serotonin in CD8 T cell antigen response, we cultured *Sert*-WT CD8 T cells in serotonin-depleted media and stimulated with anti-CD3 in the presence of SSRI (FLX or CIT; Figure 5I). SERT inhibition enhanced CD8 T cell expression of effector molecules (i.e., IL-2, IFN-γ, and Granzyme B; Figures 5J–5L, S5M, and S5N). This SSRI-induced CD8 T cell hyperactivation was abolished by blocking T cell surface 5-HTRs with the general antagonist asenapine (ASE) or by selectively inhibiting the most abundant 5-HT receptor, 5-HTR2B, using the antagonist RS-127445 (Figures 5J–5L, S5M, and S5N).^70,71^ Notably, the culture medium of SSRI-treated CD8 T cells contained significantly higher levels of serotonin after antigen stimulation, supporting an accumulation of CD8 T cell-derived serotonin (Figure 5M).

To dissect the molecular mechanisms mediating SERT regulation of CD8 T cell activation, we examined the 5-HTR/serotonin downstream signaling pathway (i.e., mitogen-activated protein kinase [MAPK]), as well as the T cell receptor (TCR)/antigen downstream signaling pathways (i.e., NFAT, nuclear factor κB [NF-κB], and activator protein 1 [AP-1]).^19,40^ SSRI treatments enhanced both 5-HTR and TCR signaling pathways, reflected by increased phosphorylation of cytoplasmic extracellular signal-regulated kinase 1/2 (ERK1/2, a component of MAPK cascade; Figure 5N), as well as increased nuclear translocation of NFAT, p65 (a component of NF-κB), and c-Jun (a component of AP-1; Figure 5O). This enhancement was largely abrogated by blocking 5-HTRs with ASE (Figures 5N and 5O). The MAPK pathway has been indicated to crosstalk with TCR signaling pathways and contribute to T cell activation.^40^ Collectively, our findings suggest that SERT negatively regulates CD8 T cell antigen responses by modulating T cell-autocrine serotonin-5-HTR-MAPK-TCR signaling.

To study whether SERT regulates intratumoral CD8 T cell-autocrine serotonin levels *in vivo*, we collected B16-OVA tumors grown in *Sert*-KO and *Sert*-WT BMT mice and measured intratumoral serotonin levels using high-performance liquid chromatography (HPLC; Figure 5P). *Sert*-KO BMT mice lacking SERT in immune cells exhibited significantly increased levels of intratumoral serotonin (Figure 5Q). In B16-OVA tumors collected from *Sert*-WT mice with or without SSRI (FLX) treatment (Figure 5S), SSRI treatment significantly elevated intratumoral serotonin levels (Figure 5T). Notably, this effect was largely abrogated by CD8 T cell depletion (Figure 5T), supporting CD8 T cells as a major contributor of intratumoral serotonin. By contrast, the systemic blood serotonin levels were greatly reduced in both *Sert*-KO BMT mice and SSRI-treated mice (Figures 5R and 5U), likely due to impaired SERT-dependent serotonin transport by platelets.^20,60^ These data suggest that CD8 T cell-intrinsic SERT plays a key role in negatively regulating the availability of local serotonin in tumors.

Together, these *in vitro* and *in vivo* data support SERT as a negative-feedback regulator restraining CD8 T cell reactivities, at least partly through directly regulating intratumoral CD8 T cell-autocrine serotonin signaling.

### SERT blockade for cancer immunotherapy: Human T cell and clinical data correlation studies

**Figure 6.**
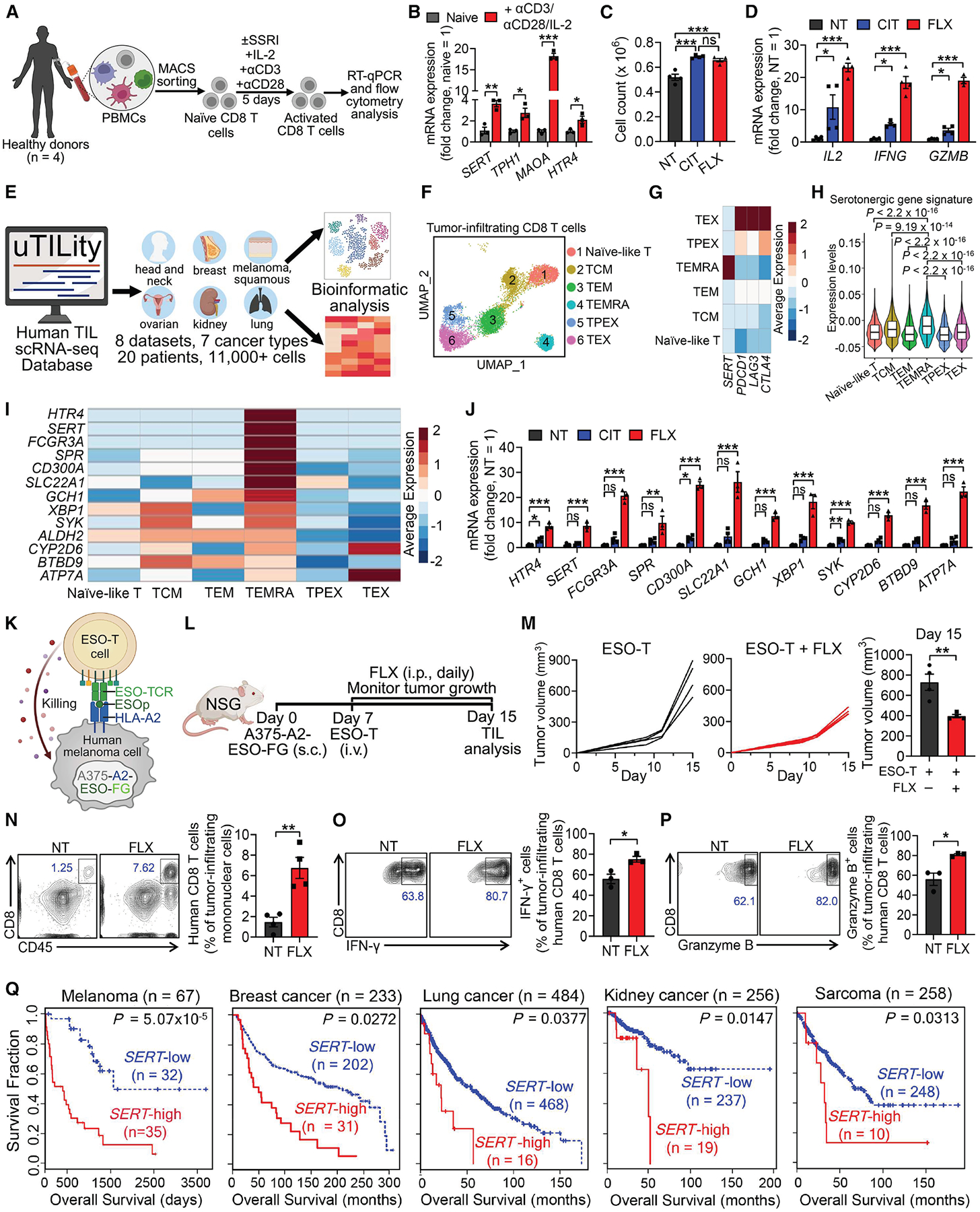
SERT blockade for cancer immunotherapy: Human T cell and clinical data correlation studies (A–D) Studying human CD8 T cell antigen responses under SSRI treatment. (A) Experimental design. Human naive CD8 T cells were sorted from healthy donor PBMCs and stimulated with anti-CD3/anti-CD28/IL-2 *in vitro* for 5 days in the absence (non-treated, NT) or presence of SSRI (FLX or CIT) treatment. PBMCs, peripheral blood mononuclear cells; αCD28, anti-CD28. (B) RT-qPCR analyses of the serotonergic gene expression in the indicated CD8 T cells at day 1 (*n* = 3). Unstimulated naive CD8 T cells were included as a control. (C) Cell counts at day 3 (*n* = 3). (D) RT-qPCR analyses of effector genes (i.e., *IL2*, *IFNG*, and *GZMB*) at day 3 (*n* = 4). (E–I) Studying the gene profile of human tumor-infiltrating CD8 T cells. (E) Experimental design. Eight scRNA-seq datasets across seven cancer types (SRA: PRJNA705464; EGA: EGAS00001004809; GEO: GSE123813, GSE164522, GSE179994, GSE181061, GSE200996, and GSE212217) were retrieved from the “uTILity” human TIL scRNA-seq database and combined for the analysis. (F) Combined UMAP plot showing the formation of six major cell clusters of human tumor-infiltrating CD8 T cells. Each dot represents a single cell and is colored according to its cell cluster assignment. TCM, central memory T; TEM, effector memory T; TEMRA, terminally differentiated effector memory CD45RA re-expressing T; TPEX, progenitor exhausted T; TEX, exhausted T. (G) Heatmap showing gene expression in the indicated CD8 T cell clusters. (H) Violin plots showing the expression distribution of the serotonergic gene signature in each CD8 T cell cluster. (I) Heatmap displaying the expression of representative serotonin pathway genes selected from the serotonergic gene signature shown in (H). (J) RT-qPCR analyses of the representative serotonin pathway genes in human CD8 T cells sorted from healthy donor PBMCs and stimulated *in vitro* without (non-treated, NT) or with SSRI (FLX or CIT) for 3 days (*n* = 3–4). (K–P) Studying SERT blockade therapy in an A375 human melanoma xenograft model. (K) Schematics showing a human tumor-T cell pair designated for this study. A375-A2-ESO-FG, human A375 melanoma cell line engineered to express the tumor antigen NY-ESO-1, its matching MHC molecule (HLA-A2), and a dual-reporter comprising a firefly luciferase and an enhanced green fluorescence protein (FG). ESO-T, human CD8 T cell engineered to express an NY-ESO-1 antigen-specific TCR. ESOp, NY-ESO-1 peptide. (L) Experimental design. (M) Tumor growth (*n* = 4). (N–P) FACS analyses of tumor-infiltrating human CD8 T cell numbers(N) and intracellular production of IFN-γ (O) and Granzyme B (P) (*n* = 3–4). (Q) Clinical data correlation studies. Kaplan-Meier plots are presented, showing the association between *SERT* expression in tumor and survival of cancer patients in a melanoma cohort (Prediction of Clinical Outcomes from Genomic Profiles [PRECOG]: GSE8401, *n* = 67), a breast cancer cohort (Molecular Taxonomy of Breast Cancer International Consortium [METABRIC], *n* = 233), a lung cancer cohort (The Cancer Genome Atlas [TCGA], *n* = 484), a kidney cancer cohort (TCGA, *n* = 256), and a sarcoma cohort (TCGA, *n* = 258). Representative of two (B–D, J, and M–P) experiments. Data are presented as the mean ± SEM. ns, not significant, *p < 0.05, **p < 0.01, and ***p < 0.001 by Student’s t test (B and M–P), one-way ANOVA (C, D, and J), or two-sided Wald test in a Cox-PH regression (Q). *p* values of violin plots were determined by the Kruskal-Wallis test for the overall comparison and Dunn’s test for post hoc pairwise comparisons between groups (H). See also Figure S6.

To explore the translational potential of SERT blockade therapy, we first studied SERT regulation of human CD8 T cell antigen responses. Human naive CD8 T cells isolated from peripheral blood mononuclear cells (PBMCs) of healthy donors were stimulated with anti-CD3/anti-CD28 *in vitro* in the presence of an SSRI (CIT or FLX; Figure 6A). In response to antigen stimulation, human CD8 T cells showed a significant increase in gene expression of key serotonergic molecules (i.e., *SERT*, *TPH1*, and *MAOA*; Figure 6B), resembling our findings in mouse CD8 T cells. Thirteen 5-HTR subtypes are known to be functional in human cells.^72^ We found that only the *HTR4* gene was consistently expressed in activated CD8 T cells across different donors (Figure S6A), and this gene was also upregulated by antigen stimulation (Figure 6B). Of note, SSRI-treated CD8 T cells showed enhanced proliferation (Figure 6C) and effector functions, characterized by upregulated expression of effector cytokines (i.e., IL-2, IFN-γ, and TNF-α; Figures 6D and S6B–S6D) and cytotoxic molecules (i.e., Granzyme B and Perforin; Figures 6D, S6E, and S6F).

To study the serotonergic gene profile in human tumor-infiltrating CD8 T cells, we analyzed eight datasets across seven cancer types from the “uTILity” human tumor-infiltrating lymphocyte (TIL) scRNA-seq database (Figure 6E; GitHub: https://github.com/ncborcherding/utility).^73^ UMAP analysis of combined human CD8 TILs identified six T cell clusters (Figure 6F): naive-like, central memory (TCM), effector memory (TEM), terminally differentiated effector memory CD45RA re-expressing (TEMRA), progenitor exhausted (TPEX), and exhausted (TEX).^74,75^ Interestingly, we observed sharp upregulation of *SERT* (Figure 6G) and an overall enhancement of the serotonergic gene profile (Figures 6H and 6I) in the TEMRA cell cluster compared with other clusters. CD8 TEMRA cells are considered as the most potent cytotoxic T cells against tumors, and increased frequencies of peripheral TEMRA cells are positively associated with ICB response.^76–78^ Notably, genes encoding classical immune checkpoints such as *PDCD1*, *LAG3*, and *CTLA4* showed a distinct expression pattern and were highly upregulated in TEX cells but not in TEMRA cells (Figure 6G). When we stimulated human CD8 T cells in the presence of an SSRI (FLX or CIT) *in vitro*, we observed a dramatic induction of a large set of serotonin pathway-related genes (Figure 6J), consistent with their upregulation in TEMRA cells (Figure 6I). These results suggest SERT blockade using SSRIs may benefit the generation and maintenance of CD8 TEMRA cells, thereby providing an avenue to enhance T cell-based cancer immunotherapy.^76–78^

To evaluate whether SSRI treatment enhances human CD8 T cell antitumor responses *in vivo*, we utilized a pre-established human melanoma xenograft NSG mouse model.^19^ We engineered the A375 human melanoma cell line to co-express tumor antigen New York esophageal squamous cell carcinoma 1 (NY-ESO-1), its matching major histocompatibility complex (MHC) molecule, human leukocyte antigen serotype A2 (HLA-A2), and an FG dual-reporter comprising a firefly luciferase and an enhanced green fluorescence protein (denoted as A375-A2-ESO-FG).^19^ In addition, CD8 T cells from healthy donor PBMCs were engineered to express an NY-ESO-1-specific TCR (denoted as ESO-TCR; Figure S6G).^19^ Thus, these CD8 T cells (denoted as ESO-T cells) can specifically target the A375-A2-ESO-FG melanoma cells (Figure 6K).^19^

We challenged NSG mice with the A375-A2-ESO-FG cells and adoptively transferred the ESO-T cells on day 7, followed by daily SSRI (FLX) treatment (Figure 6L). SSRI treatment significantly suppressed tumor growth (Figure 6M) and markedly increased the abundance of intratumoral CD8 T cells (Figure 6N). These T cells were accompanied by enhanced effector function characterized by elevated intracellular production of IFN-γ (Figure 6O), Granzyme B (Figure 6P), and Perforin (Figure S6H). Tumor suppression was not observed in NSG mice that received SSRI treatment without adoptive ESO-T cell transfer (Figures S6I and S6J).

Human melanoma cells produce minimal serotonin,^79^ whereas certain solid tumors, classified as neuroendocrine tumors (NETs), can produce serotonin.^80,81^ We then investigated the therapeutic potential of SSRIs in a PC3 human prostate neuroendocrine cancer xenograft model.^81^ The PC3 human prostate cancer cell line was similarly engineered to co-express NY-ESO-1, HLA-A2, and the FG dual-reporters (denoted as PC3-A2-ESO-FG; Figure S6K).^82^ The previously described ESO-T cells were used to model the anti-PC3 human CD8 T cell response (Figure S6K). NSG mice were inoculated with PC3-A2-ESO-FG cells, followed by ESO-T cell adoptive transfer and SSRI treatment (Figure S6L). We found that SSRI treatment almost completely suppressed the PC3 tumor growth (Figure S6M). Further analyses revealed a dramatic increase in both the abundance (Figure S6N) and effector function (i.e., IL-2 production; Figure S6O) of intratumoral ESO-T cells. Importantly, tumor suppression was absent in NSG mice receiving only the SSRI treatment without ESO-T cell adoptive transfer (Figures S6P and S6Q). These findings are intriguing, highlighting the promising potential of SSRIs in targeting NETs, which are notorious for their resistance to standard treatments and existing ICB therapies.^83^

To investigate the clinical relevance of *SERT*, we conducted a correlation analysis of *SERT* gene expression in whole-tumor lysate transcriptome data with clinical patient outcomes by using the tumor immune dysfunction and exclusion (TIDE) computational method.^84^ We observed that intratumoral *SERT* expression levels were negatively correlated with patient survival in a broad range of solid cancers, spanning melanoma, breast cancer, lung cancer, kidney cancer, and sarcoma (Figure 6Q).

Together, the human T cell and clinical correlation data support our model of SERT-mediated negative regulation of human antitumor CD8 T cell immunity and position SSRI antidepressants as promising candidates for next-generation cancer immunotherapy.

## DISCUSSION

**Figure 7.**
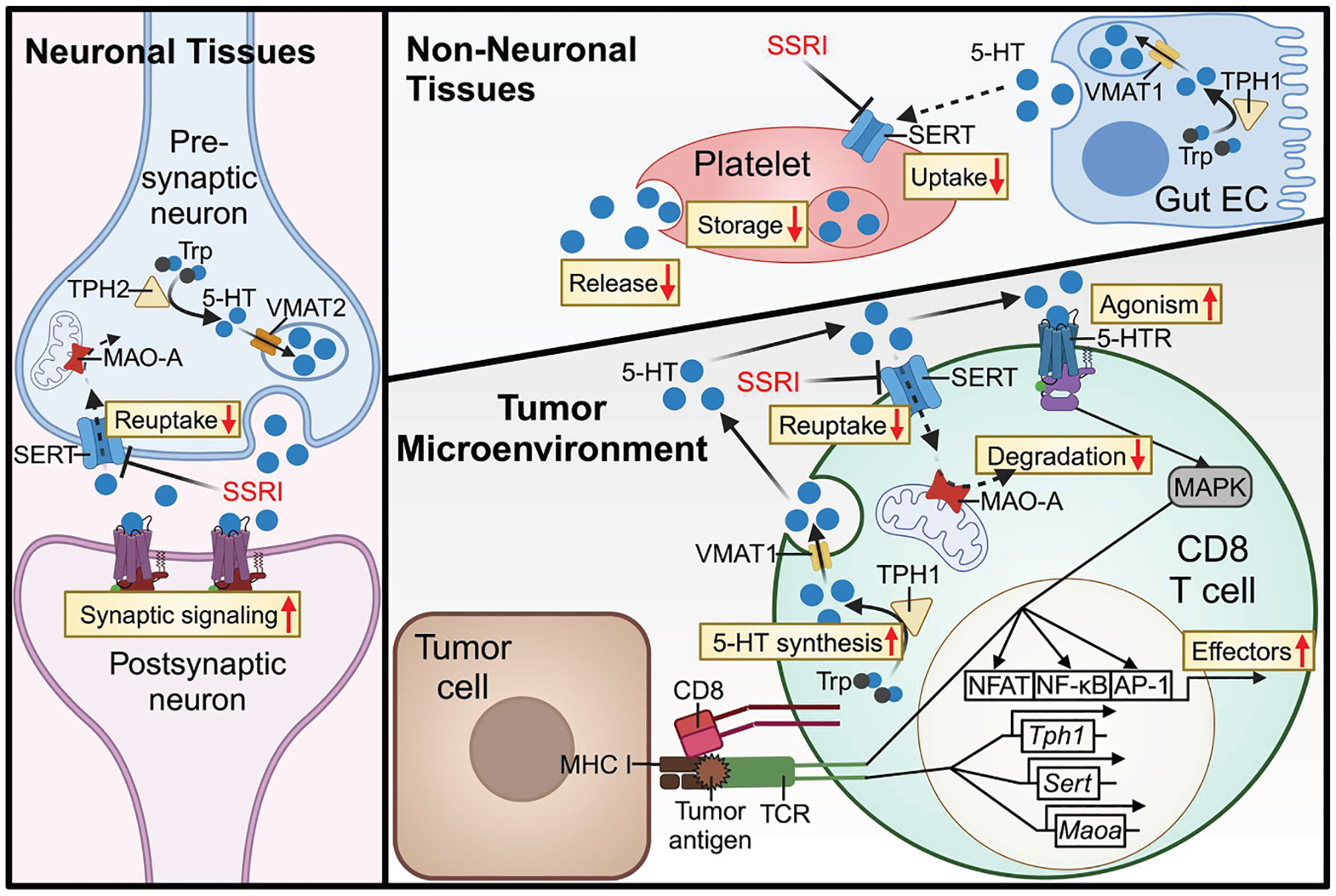
Working model of intratumoral serotonin axis regulation of CD8 T cell anti-tumor immunity Schematics are presented, showing an intratumoral serotonin axis regulation of CD8 T cell antitumor immunity. In this model, SERT restrains CD8 T cell antitumor responses by inhibiting the CD8 T cell-autocrine 5-HT signaling pathway in a solid tumor. CD8 T cells are major producers of 5-HT (or serotonin) in the tumor microenvironment (TME). Upon recognition of tumor antigen, tumor-infiltrating CD8 T cells upregulate TPH1, which synthesizes 5-HT followed by releasing it into the TME to enhance T cell activation via 5-HT signaling. Meanwhile, tumor-infiltrating CD8 T cells also upregulate SERT, which acts in a negative-feedback loop to downregulate T cell activation by terminating 5-HT signaling via the reuptake of extracellular 5-HT from the TME. Blocking SERT activity using established SSRI antidepressants accumulates 5-HT in the TME, leading to the activation of the 5-HTR-MAPK-TCR signaling pathway and enhancement of CD8 T cell antitumor reactivities. This local serotonin accumulation induced by SSRIs resembles the effect observed in neuronal tissues, where SSRIs block the reuptake of serotonin by the presynaptic neuron, thereby accumulating serotonin secreted by the presynaptic neuron and stimulating neuronal activity. Note SSRIs exhibit different effects on systemic serotonin, where SSRIs block platelet uptake of gut enterochromaffin cell (EC)-produced serotonin and deplete serum serotonin.

The nervous and immune systems possess extensive molecular overlap, with many molecules traditionally considered “neuronal” or “immune” playing key roles in both systems.^85–89^ Recently, the bidirectional crosstalk between these systems has gained increasing interest in tumor immunology.^90–92^ Notably, beyond neuroimmune crosstalk, traditional neurotransmitters have been shown to play a neuron-independent role in intratumoral signaling.^18,92,93^ Previously, our group identified MAO-A, which degrades monoamine neurotransmitters such as serotonin, as a modulator of antitumor immune responses.^18,19,54,94^ Here, we define the role of the intratumoral serotonin axis in shaping CD8 T cell antitumor immunity and establish SERT as a potent negative regulator and promising immune checkpoint target (Figure 7). Compared with its role in the nervous system, SERT regulation in intratumoral T cells differs in three key aspects: (1) highly dynamic expression, (2) an autocrine and paracrine serotonin axis, and (3) distinct 5-HT receptor signaling (Table S2).^41,42^ These findings provide fundamental insights into the molecular network governing T cell antitumor immunity and could guide the development of next-generation cancer immunotherapy.

Our previous work demonstrated that MAOI antidepressants, which inhibit intracellular monoamine degradation, significantly enhance antitumor immunity.^19,54^ However, MAOI clinical use is limited by severe side effects, including serotonin syndrome and hypertensive crises, due to extensive drug interactions and non-selective MAO inhibition.^95–98^ By contrast, SSRIs selectively target the SERT, avoiding interference with other monoaminergic pathways.^96^ This specificity contributes to their favorable safety profile, making them the most widely prescribed antidepressants.^35^ In this study, SSRI treatment exhibited similarly potent, yet slightly enhanced, antitumor efficacy compared with MAOI treatment (Figures S7A and S7B). Additionally, SSRI-treated intratumoral CD8 T cells showed upregulation of similar signaling pathways (Figures 2 and S7C–S7G). Unlike MAOIs, which induced aggressive behaviors in mice, SSRIs did not cause abnormal behaviors or adverse immune activation (Figure S1).^94^ These findings highlight SSRIs as safer, more effective candidates for targeting the intratumoral serotonin axis in next-generation cancer immunotherapy.

The best-known function of serotonin in regulating inflammation is within the gut mucosa.^51,99,100^ Studies using inflammatory bowel disease (IBD) mouse models have reported that SERT dysfunction exaggerates enteric inflammation through increased mucosal serotonin.^101–104^ Emerging evidence also highlights peripheral serotonin’s role in promoting inflammatory chemotaxis and immune cell activation (e.g., macrophages, DCs, and T cells).^40,50,68,105^ Notably, CD8 T cells possess the complete molecular machinery for serotonin regulation,^40–42^ most components of which are upregulated upon antigen stimulation (Figures 1, 5, 6, and S5). Our study identified tumor-infiltrating CD8 T cells as the primary producers and mediators of a local, immunomodulatory serotonin axis independent of the gut, providing a working model in which SERT-regulated intratumoral serotonin modulates CD8 T cell immunity within the TME.

Although serotonin has been implicated in *in vitro* tumor cell proliferation,^106–109^ its comprehensive effects on tumor cells and the microenvironment remain ambiguous. Serotonin can function as an extracellular signal through the agonism of G protein-coupled and ligand-gated ion channel 5-HTRs or as an intracellular signal through transglutaminase 2 (TGM2)-mediated serotonylation.^20,110^ Both processes have been linked to increased ERK phosphorylation, which drives Yes-associated protein (YAP)-induced cancer cell proliferation.^108,109^ Agonism of 5-HTR2B has also been shown to sustain liver cancer cell survival via phosphorylation of mTOR and enhance proliferation via Notch signaling.^111,112^ However, serotonin deficiency (*Tph1*^−/−^) in mice has been shown to diminish TAM-mediated angiogenesis and decrease tumor cell PD-L1 expression, thereby compromising tumor resources and enhancing immunogenicity.^113,114^ Notably, our study revealed that SSRI treatment directly activated CD8 T cell responses via a T cell-autocrine serotonin-5-HTR-MAPK-TCR signaling pathway (Figures 4 and 5). In NSG mice lacking mature lymphocytes, SSRI treatment did not affect the *in vivo* growth of mouse or human tumor cells (Figures S3 and S6). These findings highlight an immune-intrinsic role of serotonin in promoting anti-tumor responses.

ICB therapy benefits only a fraction of cancer patients, highlighting the need to identify additional nonredundant checkpoints to improve efficacy and broaden therapeutic applicability.^17,115^ In our study, SSRI-treated intratumoral T cells exhibited a distinct set of upregulated proliferation/effector-related pathways compared with anti-PD-1-treated T cells (Figures 2 and S2). Additionally, SSRI treatment predominantly targeted the effector/proliferating CD8 T subset, unlike anti-PD-1 treatment, which primarily sustains progenitor exhausted tumor-infiltrating CD8 T cells (Figures 2 and S2).

Analysis of existing human clinical data revealed that serotonergic regulatory genes were most highly expressed in CD8 TEMRA cells (Figure 6), a subset known for potent antitumor cytotoxicity and favorable prognosis.^60,76–78,116–118^ By contrast, the targets of approved ICB therapies were most highly expressed in exhausted CD8 T cells (Figure 6), further suggesting that different CD8 T subsets may benefit from SERT inhibition and classical ICB therapies. Additionally, a serotonylation-dependent pathway enhanced expression of PD-L1 on cancer cells and restricted tumor immunogenicity,^114^ suggesting a possible tumor-intrinsic mechanism by which SSRI treatment enhances anti-PD-1 therapy efficacy. Together, these findings suggest multifaceted, nonredundant mechanisms driving the antitumor effects of SSRIs and anti-PD-1, supporting the observed synergy of combination therapy.

The widespread prescription of antidepressants and the co-occurrence of depression in cancer patients provide an opportunity for clinical studies investigating correlations between antidepressant use and cancer patient survival.^119,120^ A nationwide cohort study of 42,075 Israeli cancer patients reported that adherence to a prescribed antidepressant at a rate above 50% was associated with one-quarter less mortality over 4 years compared with adherence below 20%.^121^ Similarly, a large-cohort clinical study from Taiwan reported a dose-dependent relationship between antidepressant use and overall survival in patients with gastric cancer after surgery and adjuvant chemotherapy.^122^ Although these studies did not discriminate between antidepressant classes, SSRIs likely drove the observed effects, as they comprised over 60% of prescriptions in 2015.^35^ Our clinical correlation analyses further highlighted the clinical significance of SERT, as high intratumoral SERT expression correlates with poor clinical outcomes in a broad range of cancers (Figure 6).

In conclusion, we identify SERT as an immune checkpoint negatively regulating CD8 T cell antitumor immunity through modulating intratumoral T cell-autocrine serotonin and demonstrate the potential of targeting the intratumoral serotonin axis using SSRI antidepressants for T cell-based cancer immunotherapy. Our findings provide a rational mechanistic basis for investigating the clinical effects of SSRIs on CD8 T cell antitumor immunity and examining the potential benefits of combining SSRIs with existing ICB therapies in cancer patients. Given the widespread clinical use of SSRIs, these strategies can be readily translated into clinical trials. Additionally, the SERT-regulated serotonergic pathway is one of several neuro-regulatory pathways only beginning to be understood as immunomodulatory within tumors. Future studies are encouraged to uncover additional neuronal regulatory genes and neurotransmitters in tumor immunology, which could further elucidate the complex regulatory networks governing antitumor immunity.

### Limitations of the study

Our study presents a negative correlation between SERT expression and patient survival and demonstrates that SSRIs can activate human CD8 T cells and promote antitumor immunity *in vitro* and *in vivo*; however, clinical correlation data linking SSRI use to overall survival/ICB response in cancer patients remain lacking. As many cancer patients suffer from depression and anxiety and may receive ICB and SSRI treatments,^119^ follow-up studies analyzing clinical outcomes associated with SSRI use in this context are required. Additionally, SERT may regulate other immune cells within the TME. Several types of immune cells (e.g., macrophages, DCs, and Treg cells) express the serotonergic machinery.^44,123^ The mechanisms by which SERT regulates the immune responses of these cells in the TME remain incompletely addressed and should be the focus of future studies.^54^ Similarly, serotonin is an important signaling molecule in many systems, particularly in the brain.^23,110^ While this study focused on immune-intrinsic serotonergic regulation, it is undefined whether SSRIs also affect intratumoral neurogenesis and neuroimmune crosstalk that can influence disease progression.^59,124,125^ Further investigation into these cross-system complexities of serotonin signaling and the impact of SERT inhibition on these dynamics will increase the applicability of SSRIs and deepen our understanding of the TME.

## RESOURCE AVAILABILITY

### Lead contact

Further information and requests for resources and reagents should be directed to and will be fulfilled by the lead contact, Lili Yang (liliyang@ucla.edu).

### Materials availability

Viral constructs and cell lines generated in this study are available upon signing a material transfer agreement with UCLA.

### Data and code availability

All data associated with this study are present in the paper or supplemental information. The scRNA-seq data generated in this study have been deposited at the Genome Expression Omnibus (GEO) as GEO: GSE262781 and GSE286271 and are publicly available as of the publication date. The scRNA-seq data for the human CD8 tumor-infiltrating lymphocyte atlas are available from N. Borcherding’s “uTILity” repository (GitHub: https://github.com/ncborcherding/utility).

Code and any additional information required to reanalyze the data reported in this paper are available from the lead contact upon request.

## STAR★METHODS

### EXPERIMENTAL MODEL AND STUDY PARTICIPANT DETAILS

#### Mice

C57BL/6J (B6), BALB/cJ (BALB/c), B6.SJL-*Ptprc*^*a*^*Pepc*^*b*^/BoyJ (CD45.1), C57BL/6-Tg (TcraTcrb)1100Mjb/J (*OT1*-Tg), B6.129(Cg)-*Slc6a4*^*tm1Kpl*^/J (*Sert*-KO), B6.129-*Htr7*^*tm1Sut*^/J (*5Htr7*-KO), and NOD.Cg-*Prkdc*^*scid*^ *Il2rg*^*tm1Wjl*^/SzJ (NOD *scid* gamma or NSG) mice were purchased from the Jackson Laboratory (JAX; Bar Harbor). *Sert*-KO and *5Htr7*-KO mice were backcrossed with C57BL/6J mice for more than six generations at the University of California, Los Angeles (UCLA). The *OT1*-Tg mice deficient of SERT (*OT1*-Tg/*Sert*-KO) were generated at UCLA through breeding *OT1*-Tg mice with *Sert*-KO mice. All animals were maintained in the animal facilities at UCLA. Eight- to twelve-week-old female mice were used for all experiments unless otherwise indicated. All animal experiments were approved by the Institutional Animal Care and Use Committee of UCLA.

#### Syngeneic mouse tumor models

B16-OVA melanoma cells (1 × 10^6^ per animal), B16-F10 melanoma cells (1 × 10^6^ per animal), MC38 colon cancer cells (5 × 10^5^ per animal), MB49 bladder cancer cells (2 × 10^5^ per animal), CT26 colon cancer cells (5 × 10^6^ per animal), or 4T1 breast cancer cells (1 × 10^6^ per animal) were subcutaneously injected into experimental mice to form solid tumors. For the 4T1 mouse breast cancer orthotopic model, 4T1 breast cancer cells (1 × 10^4^ per animal) were injected into the mammary fat pad of experimental female BALB/c mice to form orthotopic breast tumors. For SSRI treatment experiments, mice received intraperitoneal injection of SSRI [i.e., fluoxetine (10mg/kg/day), or citalopram (30mg/kg/day)] to block SERT activity. For MAOI treatment experiments, mice received intraperitoneal injection of MAOI [i.e., phenelzine (30 mg/kg per day)] to block MAO-A activity. For T cell depletion experiments, mice received intraperitoneal injection of anti-mouse CD8 antibody (200 mg per animal, twice per week) to deplete CD8 T cells; mice that received intraperitoneal injection of rat immunoglobulin G2b (IgG2b) isotype antibody (200 mg per animal, twice per week) were included as a control. For PD-1 blockade experiments, mice received intraperitoneal injection of anti-mouse PD-1 antibody (300 mg per animal, twice per week) to block PD-1; mice that received intraperitoneal injection of rat IgG2a isotype antibody (300 mg per animal, twice per week) were included as a control. Throughout the course of an experiment, tumor size was measured twice per week by using a Fisherbrand Traceable digital caliper (Thermo Fisher Scientific); tumor volumes were calculated by formula 1/2 × L × W^2^. At the end of an experiment, solid tumors, peripheral tissues, and blood were collected for downstream analysis.

#### Bone marrow (BM) transfer B16-OVA tumor model

BM cells were collected from femurs and tibias of the *Sert*-WT or *Sert*-KO donor mice and were transferred into the B6 recipient mice through intravenous (i.v.) injection (10 × 10^6^ cells per recipient mouse). Recipient mice were preconditioned with whole-body irradiation (1,100 rads). After BM transfer, recipient mice were maintained on antibiotic water (Amoxil; 0.25 mg/ml) for 4 weeks. Periodic bleedings were performed to monitor immune cell reconstitution using flow cytometry. At 8 to 12 weeks after BM transfer, recipient mice were fully immune-reconstituted and were used for B16-OVA mouse melanoma challenge experiments. Tumor growth was monitored twice per week by measuring tumor size using a Fisherbrand Traceable digital caliper; tumor volumes were calculated by formula 1/2 × L × W^2^. At the end of an experiment, solid tumors were collected and tumor-infiltrating immune cells (TIIs) were isolated for analysis using flow cytometry.

#### Adoptive OT1 T cell transfer B16-OVA tumor model

Spleen and lymph node cells were harvested from the *OT1*-Tg or *OT1*-Tg/*Sert*-KO mice and were subjected to magnetic-activated cell sorting (MACS) using a Mouse CD8 T Cell Isolation Kit (catalog no. 130–104-075, Miltenyi Biotec) following the manufacturer’s instructions. The purified OT1 T cells (identified as CD8^+^TCR Vβ5^+^ cells) were adoptively transferred to tumor-bearing CD45.1 wild-type mice (1 × 10^5^ cells per recipient mouse). CD45.1 mice were subcutaneously inoculated with B16-OVA tumor cells 1 week in advance (1 × 10^6^ cells per animal). Before OT1 T cell adoptive transfer, recipient mice were preconditioned with whole-body irradiation (600 rads). During an experiment, tumor growth was monitored twice per week by measuring tumor size using a Fisherbrand Traceable digital caliper; tumor volumes were calculated by formula 1/2 × L × W^2^. Mice were terminated at the indicated time points, and solid tumors were collected and TIIs were isolated for analysis using flow cytometry.

#### Xenograft human tumor models

The A375-A2-ESO-FG human melanoma cells (10 × 10^6^ cells per animal) or the PC3-A2-ESO-FG neuroendocrine human prostate cancer cells (5 × 10^6^ cells per animal) were subcutaneously injected into NSG mice to form solid tumors. Mice received fluoxetine treatment through intraperitoneal injection (10 mg/kg/day). In some experiments, mice received ESO-T cells through intravenous injection (10 × 10^6^ cells per recipient mouse). During an experiment, tumor growth was monitored twice per week by measuring tumor size using a Fisherbrand Traceable digital caliper; tumor volumes were calculated by formula 1/2 × L × W^2^. At the end of an experiment, solid tumors were collected and TIIs were isolated for analysis using flow cytometry.

#### Tumor cell lines

The B16-OVA mouse melanoma cell line and the PG13 retroviral packaging cell line were kindly provided by Dr. Pin Wang (University of Southern California, CA, USA). The MC38 mouse colon adenocarcinoma cell line was provided by Dr. Marcus Bosenberg (Yale University, CT, USA). The MB49 mouse bladder cancer cell line was provided by Dr. Arnold Qin (University of California, Los Angeles, CA, USA). The human embryonic kidney 293T, PC3 human prostate cancer, B16-F10 mouse melanoma, CT26 mouse colon cancer, and 4T1 mouse breast cancer cell lines were purchased from the American Type Culture Collection (ATCC). The A375-A2-ESO-FG human melanoma cell line^19^ and the PC3-A2-ESO-FG human prostate cancer cell line^82^ were generated by our group. 4T1 and CT26 tumor cell lines were cultured in an R10 medium as described below. All other tumor cell lines were cultured in the D10 medium as described below.

#### Healthy donor peripheral blood mononuclear cells (PBMCs)

Healthy donor PBMCs were purchased from the UCLA Center for AIDS Research (CFAR) Virology Core Laboratory without identification information under federal and state regulations.

### METHOD DETAILS

#### Media and reagents

DMEM-based adherent cell culture medium (denoted as D10 medium) was made of Dulbecco’s modified Eagle’s medium (DMEM; catalog no. 10013, Corning) supplemented with 10% fetal bovine serum (FBS; catalog no. F2442, Sigma-Aldrich) and 1% penicillin-streptomycin-glutamine (catalog no. 10378016, Gibco). RPMI-based adherent cell culture medium (denoted as R10 medium) was made of RPMI 1640 (catalog no. 10040, Corning) supplemented with 10% fetal bovine serum (FBS; catalog no. F2442, Sigma-Aldrich) and 1% penicillin-streptomycin-glutamine (catalog no. 10378016, Gibco). T cell culture medium (denoted as C10 medium) was made of RPMI 1640 (catalog no. 10040, Corning) supplemented with 10% FBS (catalog no. F2442, Sigma-Aldrich), 1% penicillin-streptomycin-glutamine (catalog no. 10378016, Gibco), 0.2% Normocin (catalog no. ant-nr-2, InvivoGen), 1% Minimal Essential Medium (MEM) Non-essential Amino Acid Solution (catalog no. 11140050, Gibco), 1% HEPES (catalog no. 15630080, Gibco), 1% sodium pyruvate (catalog no. 11360070, Gibco), and 0.05 mM β-mercaptoethanol (catalog no. M3148, Sigma-Aldrich).

Cell culture reagents—including purified no azide/low endotoxin (NA/LE) anti-mouse CD3ε (catalog no. 553057, clone 145–2C11), purified NA/LE anti-mouse CD28 (catalog no. 553294, clone 37.51), and GolgiStop (catalog no. 554724)—were purchased from BD Biosciences. Anti-human CD3 (catalog no. 300314, clone HIT3a) and anti-human CD28 (1 μg/ml; catalog no. 302902, clone CD28.2) were purchased from BioLegend. Recombinant human IL-2 (catalog no. 200–02) was purchased from PeproTech. L-Ascorbic acid (100 μM; catalog no. A4403), phorbol-12-myristate-13-acetate (PMA) (catalog no. 524400), ionomycin (catalog no. I0634), and charcoal-dextran (catalog no. C6241) were purchased from Sigma-Aldrich.

*In vivo* depletion antibodies, including anti-mouse CD8α (catalog no. BE0061, clone RMP2.43) and its isotype control [rat immunoglobulin G2b (IgG2b), catalog no. BE0090], were purchased from BioXCell. *In vivo* PD-1–blocking antibody (catalog no. BE0146, clone RMP1–14) and its isotype control (rat IgG2a, catalog no. BE0089) were purchased from BioXCell.

SSRIs—including fluoxetine (catalog no. ab120077) and citalopram (catalog no. ab120133)—were purchased from Abcam. An MAOI, phenelzine (catalog no. P6777), was purchased from Sigma-Aldrich. Serotonin (catalog no. H9532), a general serotonin receptor (5-HTR) antagonist asenapine (ASE, catalog no. A7861), and a selective 5-HTR2B antagonist RS-127445 (catalog no. R2533) were also purchased from Sigma-Aldrich.

#### Tumor-infiltrating immune cell (TII) isolation and *ex vivo* analysis

Solid tumors were harvested from experimental mice and mechanically meshed in 70-μm cell strainers (catalog no. 07-201-431, Corning) to generate single cell suspensions. Single cells were washed once with the C10 medium, resuspended in 50% Percoll (catalog no. P4937, Sigma-Aldrich), and centrifuged at 800g at 25°C for 30 min with brake off. Cell pellets enriched with TIIs were then collected for further analysis.

In the experiments studying gene expression in tumor-infiltrating CD8 T cell subsets, day 14 B16-OVA tumors were harvested from the B6 wild-type mice to prepare TII suspensions. Tumor-infiltrating CD8 T cells (pregated as DAPI^−^CD45.2^+^ TCRβ^+^CD8^+^ cells) were sorted into two subsets (gated as PD-1^lo^ and PD-1^hi^Tim-3^hi^LAG-3^hi^ cells) using a FACSAria II flow cytometer and then were subjected to qPCR analysis.

In the experiments studying gene expression profiles of TIIs, day 10 B16-OVA tumors were harvested from the SSRI-treated and anti-PD-1-treated mice to prepare TII suspensions. TII suspensions were then sorted using a FACSAria II flow cytometer to purify immune cells (gated as DAPI^−^CD45.2^+^ cells) that were subjected to scRNA-seq analysis.

In the experiments studying status and function of the tumor-infiltrating CD8 T cells, TII suspensions were prepared and then analyzed by flow cytometry to study their expression of surface activation/exhaustion markers and intracellular effector molecules.

#### Histological analysis

For histological sectioning, organs (e.g. heart, lung, kidney, spleen, and liver) were harvested from the experimental mice, placed into 10% neutral-buffered formalin (catalog no. 5705, Richard-Allan Scientific) immediately, fixed for 18 h, and then transferred to 70% ethanol before standard paraffin embedding for sectioning (5-μm thickness), followed by hematoxylin and eosin (H&E) staining using standard procedures (UCLA Translational Pathology Core Laboratory). The sections were photographed using an upright microscope (BX-51; Olympus) and using a color charge-coupled device digital camera (Insight 4 MP; SPOT) and software (SPOT).

#### *In vitro* mouse CD8 T cell culture

Spleen and lymph node cells were harvested from the *Sert*-WT and *Sert*-KO mice. Naive CD8 T cells were sorted using a Mouse Naïve CD8 T Cell Isolation Kit (catalog no. 130-096-543, Miltenyi Biotec) according to the manufacturer’s instructions or using a FACSAria II flow cytometer (gated as DAPI^−^TCRβ^+^CD8^+^CD44^lo^CD62L^hi^ cells). Purified mouse naïve CD8 T cells were cultured *in vitro* in C10 medium in a 24-well plate at 0.5 × 10^6^ cells per ml per well, in the presence of plate-bound anti-mouse CD3 (5 μg/ml) with or without anti-mouse CD28 (1 μg/ml) as T cell stimulators for up to 4 days. At indicated time points, cells were collected for quantitative reverse-transcription PCR (RT-qPCR) analysis of gene expression, and cell culture supernatants were collected for ELISA analysis of effector cytokine production. For the analysis of surface markers and intracellular cytotoxic molecule production, CD8 T cells were collected and then directly subjected to flow cytometry analysis. For the analysis of intracellular cytokine production, CD8 T cells were restimulated with PMA (50 ng/ml) and ionomycin (500 ng/ml) in the presence of GolgiStop (4 μl per 6 ml culture) for 4 hours at 37°C, followed by flow cytometry analysis.

In the experiments studying the effects of SSRIs, purified *Sert*-WT CD8 T cells were also treated with either fluoxetine (2 μM) or citalopram (20 μM). In the experiments studying autocrine serotonin signaling, cells were cultured in C10 medium made of the FBS that was pretreated overnight with charcoal-dextran (1 g per 50 ml of FBS; catalog no. C6241, Sigma-Aldrich) to deplete serotonin. L-Ascorbic acid (100 μM) was also added to the medium to stabilize the T cell-produced serotonin. In some experiments, purified CD8 T cells were also treated with the general 5-HTR antagonist ASE (10 μM) or the selective 5-HTR2B antagonist RS-127445 (1 μM) to block serotonin receptor signaling.

#### *In vitro* human CD8 T cell culture

Naive CD8 T cells were sorted from PBMCs of healthy donors using a Human Naïve CD8 T Cell Isolation Kit (catalog no. 130-093-244, Miltenyi Biotec) according to the manufacturer’s instructions. Human naive CD8 T cells were then cultured *in vitro* in a 24-well plate at0.5 × 10^6^ cells per well in T cell culture medium in the presence of plate-bound anti-human CD3 (5 μg/ml), soluble anti-human CD28 (1 μg/ml) and soluble recombinant human IL-2 (10 ng/ml) for up to 5 days. In the experiments studying the effects of SSRIs, human naive CD8 T cells were treated with either fluoxetine (2 μM) or citalopram (20 μM). At indicated time points, cells were collected to analyze gene expression using RT-qPCR, or/and to analyze protein expression using western blot. To assay intracellular cytotoxicity molecule production, effector CD8 T cells were directly subjected to flow cytometry analysis. To assay intracellular cytokine production, CD8 T cells were restimulated with PMA (50 ng/ml) and ionomycin (500 ng/ml) in the presence of GolgiStop (4 μl per 6 ml culture) for 4 hours at 37°C, followed by flow cytometry analysis.

#### *In vitro* OT1 T cell culture

Spleen and lymph node cells were harvested from the *OT1*-Tg or *OT1*-Tg/*Sert*-KO mice and then subjected to MACS sorting using a Mouse CD8 T Cell Isolation Kit (catalog no. 130-104-075, Miltenyi Biotec) following the manufacturer’s instructions. The purified OT1 T cells (identified as CD8^+^TCR Vβ5^+^ cells) were cultured in C10 medium in a 24-well plate at 0.5 × 10^6^ cells per ml medium per well, in the presence of plate-bound anti-mouse CD3 (5 μg/ml) for up to 4 days. At the indicated time points, cells were collected for RT-qPCR analysis of effector molecule gene expression, and cell culture supernatants were collected for ELISA analysis of effector cytokine production.

#### Retro/ESO-TCR retroviral vector and human CD8 T cell transduction

The construction of the Retro/ESO-TCR retroviral vector and the transduction of human CD8 T cells have been previously reported.^19^ The Retro/ESO-TCR vector was constructed by inserting into the parental pMSGV vector a synthetic gene encoding an HLA-A2–restricted, NY-ESO-1 tumor antigen-specific human CD8 TCR (clone 3A1).^82^ VSVG-pseudotyped Retro/ESO-TCR retroviruses were generated by transfecting 293T cells following a standard calcium precipitation protocol and an ultracentrifugation concentration protocol; the viruses were then used to transduce PG13 cells to generate a stable retroviral packaging cell line producing gibbon ape leukemia virus (GaLV) glycoprotein-pseudotyped Retro/ESO-TCR retroviruses (denoted as PG13-ESO-TCR cell line). For virus production, the PG13-ESO-TCR cells were seeded at a density of 0.8 × 10^6^ cells/ml in D10 medium and cultured in a 15-cm dish (30 ml per dish) for 2 days; virus supernatants were then harvested and stored at −80°C for future use.

Healthy donor PBMCs were stimulated with plate-bound anti-human CD3 (1 μg/ml) and soluble anti-human CD28 (1 μg/ml) in the presence of recombinant human IL-2 (10 ng/ml). On day 2, cells were spin-infected with frozen-thawed Retro/ESO-TCR retroviral supernatants supplemented with polybrene (10 μg/ml; catalog no. TR-1003-G, Millipore) at 660g at 30°C for 90 min. Transduced human CD8 T cells (denoted as ESO-T cells) were expanded for another 7 to 10 days and then cryopreserved for future use. Mock-transduced human CD8^+^ T cells (denoted as Mock-T cells) were generated to serve as controls.

#### Flow cytometry

Flow cytometry was used to analyze the expression of surface and intracellular markers of T cells as well as to sort different subsets of T cells. Fluorochrome-conjugated monoclonal antibodies specific for mouse CD45.2 (clone 104), TCRβ (clone H57–597), TCR Vβ5 (clone MR9–4), CD4 (clone RM4–5), CD8 (clone 53–6.7), CD69 (clone H1.2F3), CD25 (clone PC61), CD44 (clone IM7), CD62L (clone MEL-14), LAG-3 (clone C9B7W), Tim-3 (clone RMT3–23), Granzyme B (clone QA16A02), and IFN-γ (clone XMG1.2) were purchased from BioLegend. Fluorochrome-conjugated monoclonal antibody specific for mouse TCF1/TCF7 (clone C63D9) was purchased from Cell Signaling. Fc block (anti-mouse CD16/32) (clone 2.4G2) was purchased from BD Biosciences. Monoclonal antibody specific for mouse PD-1 (clone RMP1–30) was purchased from Thermo Fisher Scientific. Fluorochrome-conjugated monoclonal antibodies specific for human CD45 (clone HI30), TCRα/β (clone IP26), CD4 (clone OKT4), CD8 (clone SK1), TCR Vβ13.1 (clone H131), CD62L (clone DREG-56), CD69 (clone FN50), CD25 (clone M-A251), PD-1 (clone EH12.2H7), IL-2 (clone MQ1–17H12), TNF-α (clone MAb11), Perforin (clone dG9), Granzyme B (clone QA16A02), and IFN-γ (clone B27) were purchased from BioLegend. Human Fc Receptor Blocking Solution (catalog no. 422302) was purchased from BioLegend. Fixable Viability Dye eFluor 506 (catalog no. 65–0866) was purchased from Thermo Fisher Scientific. OVA dextramer (catalog no. JD2163) was purchased from Immudex.

Cells were initially stained with a Fixable Viability Dye, followed by Fc receptor blocking and surface marker staining, as described previously.^19^ To detect intracellular molecules, cells were subjected to intracellular staining using a Cell Fixation/Permeabilization Kit (catalog no. 554714, BD Biosciences) following the manufacturer’s instructions. To detect nuclear molecules, cells were subjected to intracellular staining using a Foxp3/Transcription Factor Staining Kit (catalog no. 00-5523-00, eBioscience) following the manufacturer’s instructions. Stained cells were analyzed using a MACSQuant Analyzer 10 Flow Cytometer (Miltenyi Biotec). FlowJo 10 software (Tree Star) was used to analyze the data.

#### mRNA quantitative reverse-transcription PCR (mRNA RT-qPCR)

Total RNA was isolated using TRIzol Reagent (catalog no. 15596018, Thermo Fisher Scientific) and an miRNeasy Mini Kit (catalog no. 217004, QIAGEN) according to the manufacturers’ instructions. cDNA was prepared using a SuperScript III First-Strand Synthesis Supermix Kit (catalog no. 18080400, Thermo Fisher Scientific). Gene expression was measured using a SsoAdvanced Universal SYBR Green Supermix (catalog no. 1725271, Bio-Rad) and a 7500 Real-time PCR System (Applied Biosystems) according to the manufacturers’ instructions. *Ube2d2* was used as an internal control for mouse T cells, and *ACTIN* was used as an internal control for human T cells. The relative expression of the mRNA of interest was calculated using the 2^ΔΔCT^ method and is presented as the fold induction relative to the control. Primer sequences are shown in Table S1.

#### Enzyme-linked immunosorbent assay (ELISA)

ELISAs for detecting mouse cytokines in cell culture supernatants were performed following a standard protocol from BD Biosciences. Capture and biotinylated antibody pairs for the detection of mouse IFN-γ (coating antibody, catalog no. 554424; biotinylated detection antibody, catalog no. 554426) and IL-2 (coating antibody, catalog no. 551216; biotinylated detection antibody, catalog no. 554410) were also purchased from BD Biosciences. The streptavidin–horseradish peroxidase (HRP) conjugate (catalog no. 18410051) was purchased from Invitrogen. Mouse IFN-γ (catalog no. 575309) and IL-2 (catalog no. 575409) standards were purchased from BioLegend. The 3,3′,5,5′-tetramethylbenzidine (TMB; catalog no. 51200048) substrate was purchased from KPL. Samples were analyzed for absorbance at 450 nm using an Infinite M1000 microplate reader (Tecan).

ELISA for the analysis of T-cell produced serotonin was performed using a serotonin ultrasensitive ELISA kit (catalog no. SEU39-K01, Eagle Biosciences) following the manufacturer’s instructions. The absorbance at 450 nm was measured using an Infinite M1000 microplate reader (Tecan).

Titers of autoantibodies against double-stranded DNA were measured using a commercial mouse anti-dsDNA ELISA kit (catalog no. 637–02691, BioVendor) according to manufacturer’s instructions. The absorbance of samples at 450 nm was measured using an Infinite M1000 microplate reader (Tecan).

#### Western blot (WB)

CD8 T cells purified from the *Sert*-WT mice were cultured *in vitro* in the C10 medium in a 24-well plate at 0.5 × 10^6^ cells per ml per well for 2 days, in the presence of plate-bound anti-mouse CD3 (5 μg/ml), with or without ASE treatment (10 μM). Cells were then rested on ice for 2 hours and restimulated with plate-bound anti-mouse CD3 (5 μg/ml) for 20 min. Total protein was extracted using a RIPA lysis and extraction buffer (catalog no. 89900, Thermo Fisher Scientific) supplemented with a protease/phosphatase inhibitor cocktail (catalog no. 5872S, Cell Signaling Technology). Nuclear protein was extracted using a Nuclear Protein Extraction Kit (catalog no. P178833, Thermo Fisher Scientific). Protein concentration was measured using a Bicinchoninic Acid (BCA) Assay Kit (catalog nos. 23228 and 1859078, Thermo Fisher Scientific). Equal amounts of protein were resolved on a 10% SDS-polyacrylamide gel electrophoresis gel and then transferred to a polyvinylidene difluoride (PVDF) membrane by electrophoresis. The following antibodies were purchased from the Cell Signaling Technology and used to blot for the proteins of interest: anti-mouse NF-κB p65 (catalog no. 8242S, clone D14E12), anti-mouse c-Jun (catalog no. 9165S, clone 60A8), anti-mouse NFAT (catalog no. 4389S), anti-mouse ERK1/2 (catalog no. 9107S, clone 3A7), anti-mouse p-ERK1/2 (catalog no. 4370S, clone D13.14.4E), secondary anti-mouse (catalog no. 7076P2), and secondary anti-rabbit (catalog no. 7074P2). GAPDH (catalog no. 2118S, clone 14C10, Cell Signaling Technology) was used as an internal control for cytoplasmic proteins, while Lamin A/C (catalog no. 39287, clone 3A6–4C11, Active Motif) was used as an internal control for nuclear proteins. Signals were visualized with autoradiography using an enhanced chemiluminesence (ECL) prime western blotting system (catalog no. RPN2232, Cytiva). Data analysis was performed using ImageJ software (NIH).

#### High-performance liquid chromatography (HPLC)

HPLC was used to measure the intratumoral and serum serotonin levels as previously described.^19^ Briefly, tumor and serum samples were collected from experimental mice at indicated time points and were snap-frozen using liquid nitrogen. Frozen samples were thawed and homogenized using methanol (catalog no. 268280025, Thermo Fisher Scientific) and acetonitrile (catalog no. A998SK-1, Thermo Fisher Scientific) by vortexing. Homogenized samples were centrifuged, and supernatants were collected to new tubes and evaporated under a stream of argon. Dried sample pellets were then reconstituted in HPLC running buffer and were ready for analysis. Serotonin concentration was quantified using a C18 column by reverse-phase HPLC (System Gold 166P detector, Beckman Coulter). For tumor samples, both intracellular and interstitial serotonin were analyzed.

#### Single-cell RNA sequencing (scRNA-seq) analysis of mouse TIIs

scRNA-seq was used to analyze the gene expression profiles of mouse TIIs. Day 10 B16-OVA tumors were harvested from experimental mice to prepare TII suspensions (10 tumors were combined for each group). TII suspensions were then sorted using a FACSAria II flow cytometer to purify immune cells (gated as DAPI^−^CD45.2^+^ cells). Sorted TIIs were immediately delivered to the Technology Center for Genomics & Bioinformatics (TCGB) facility at UCLA for library construction and sequencing. Briefly, sorted TIIs were quantified using a Cell Countess II automated cell counter (Invitrogen/Thermo Fisher Scientific). A total of 10,000 TIIs from each experimental group were loaded on the Chromium platform (10x Genomics), and libraries were constructed using the Chromium Single Cell 3’ Library & Gel Bead Kit v2 (catalog no. PN-120237, 10x Genomics) according to the manufacturer’s instructions. Libraries were sequenced on an Illumina NovaSeq using a NovaSeq 6000 S2 Reagent Kit (100 cycles; catalog no. 20012862, Illumina).

For cell clustering and annotation, the merged digital expression matrix generated by Cell Ranger was analyzed using the R package Seurat (v.4.0.0) following the guidelines. Briefly, after filtering the low-quality cells, the expression matrix was normalized using the NormalizeData function, followed by selecting variable features across datasets using the FindVariableFeatures and SelectIntegrationFeatures functions. To correct for batch effects, the FindIntegrrationAnchors and IntegrateData functions were used based on the selected feature genes. The corrected dataset was subjected to standard Seurat workflow for dimension reduction and clustering. In this study, the mouse antigen-experienced tumor-infiltrating CD8 T cells (identified by co-expression of *Cd8a*, *Cd3d*, and *Cd44* marker genes) were extracted using the Subset function, and the Seurat integration pipeline was performed following the Seurat guidelines. Following integration, three clusters of CD8 T cells were manually merged and annotated using gene signatures (GEO: GSE122713) from previous studies.^126^ Similarly, clusters of mouse immune cells were merged and annotated based on immune lineage markers.^127–132^ The AddModuleScore function was used to calculate module scores of each gene signature list, while the FeaturePlot function was used to visualize the expression of each signature in the UMAP plots. Additionally, dot plots were generated using the scCustomize package (v3.0.1) in R, providing detailed information about the average expression levels of genes within clusters and the proportion of cells within each cluster that express those genes.

For gene set enrichment analysis (GSEA), the clusterProfiler packages were used to calculate the enrichment scores for each cluster in the signature gene list (GEO: GSE122713).^126^

For RNA-velocity analysis, the.LOOM files containing spliced and unspliced expression matrices were generated for each sample. Further analysis was conducted using the Velocyto.R-package (v.0.6). After loading.LOOM file information through the ReadVelocity function, databases were merged and the RunVelocity function was executed to obtain the velocity vectors. Finally, the velocities were projected into a lower-dimensional embedding using the velocity_graph function and visualized on the UMAP embedding in each intended cell cluster using the show.velocity.on.embedding.cor function. All velocity functions were used with default parameters.

Pathways were defined using gene ontology analyses.^133,134^ For pathway analysis, the fold enrichment value was calculated as following: Fold_enrichment = (N intersect / N DEG) / (N pathway / N background), where N intersect indicates the number of genes from differentially expressed gene set that are present in the pathway gene set; N DEG indicates number of genes from differentially expressed gene set; N pathway indicates the number of genes from the pathway gene set; and N background indicates the total number of genes in this analysis.

### QUANTIFICATION AND STATISTICAL ANALYSIS

#### scRNA-seq analysis of human tumor-infiltrating lymphocytes (TILs)

A publicly available dataset of tumor-infiltrating T cells (TILs) with paired TCR sequencing from N. Borcherding’s “uTILity” resource (GitHub: https://github.com/ncborcherding/utility) was utilized as the human CD8^+^ TIL atlas.^73^ This resource includes detailed information on sample processing and library preparation, providing a solid foundation for downstream analyses. The dataset comprises 11,021 high-quality single-cell transcriptomes from 20 samples spanning seven different tumor types. All subsequent analyses were performed using the Seurat package (v5.1.0) in R.

For cell clustering and annotation, the merged digital expression matrix was processed following Seurat’s established guidelines. TIL clusters were manually identified and annotated based on gene signatures reported in prior studies.^135–140^ Module scores representing the expression of specific gene signatures were calculated using the AddModuleScore function and visualized on a UMAP plot with the DimPlot function. Genes in the serotonin signaling pathway were further assessed across human TIL groups using violin plots generated with the VlnPlot function.

#### Tumor Immune Dysfunction and Exclusion (TIDE) analysis

Human clinical correlation studies were conducted by using an established TIDE computational method (http://tide.dfci.harvard.edu/query/). The survival correlation function of TIDE was used to study the clinical data correlation between the intratumoral *SERT* gene expression and patient survival. For each patient cohort, tumor samples were divided into two groups: *SERT*-high (samples with *SERT* expression one standard deviation above the average) and *SERT*-low (remaining samples) groups. The association between the intratumoral *SERT* gene expression levels and patient overall survival (OS) was assessed using a two-sided Wald test in the Cox proportional hazards (Cox-PH) regression model and presented in Kaplan–Meier plots.

#### Other statistical analyses

GraphPad Prism 9 software (GraphPad Software) was used for statistical data analysis. Pairwise comparisons were made using a two-tailed Student’s t test. Multiple comparisons were performed using an ordinary one-way analysis of variance (ANOVA) followed by Tukey’s multiple comparisons test or a two-way repeated measures ANOVA followed by Sidak multiple comparisons test. Meier survival curves analyses were performed using log rank (Mantel-Cox) test adjusted for multiple comparisons. Data are presented as the mean ± SEM, unless otherwise indicated. A *p* value of less than 0.05 was considered significant. ns, not significant; *p < 0.05, **p < 0.01, and ***p < 0.001. The *p* values of violin and GSEA plots were determined by Kruskal-Wallis test with Dunn’s test as a post-hoc test. For the Kaplan-Meier plots of overall patient survival with different *SERT* levels, the *p* values were calculated using a two-sided Wald test in the Cox-PH regression model.

## Supplementary Material

MMC1

1

Supplemental information can be found online at https://doi.org/10.1016/j.cell.2025.04.032.

## Figures and Tables

**Table:** KEY RESOURCES TABLE

REAGENT or RESOURCE	SOURCE	IDENTIFIER
Antibodies		
Anti-mouse IFN-γ (ELISA, coating)	BD Biosciences	CAT# 551216; RRID: AB_394094
Anti-mouse IFN-γ (ELISA, detection)	BD Biosciences	CAT# 554410; RRID: AB_395374
Anti-mouse IL-2 (ELISA, coating)	BD Biosciences	CAT# 554424; RRID: AB_395383
Anti-mouse IL-2 (ELISA, detection)	BD Biosciences	CAT# 554426; RRID: AB_395384
PE/Cyanine7 anti-mouse CD45.2 (Clone 104)	Biolegend	CAT# 109830; RFID: AB_1186098
APC/Cyanine7 anti-mouse TCRβ (Clone H57-597)	Biolegend	CAT# 109220; RRID: AB_893624
PE anti-mouse TCR Vβ5 (Clone MR9-4)	Biolegend	CAT# 139503; RRID: AB_10613279
FITC anti-mouse CD4 (Clone RM4-5)	Biolegend	CAT# 100510; RRID: AB_312713
PerCP anti-mouse CD8a (Clone 53-6.7)	Biolegend	CAT# 100732; RRID: AB_893423
APC anti-mouse CD69 (Clone H1.2F3)	Biolegend	CAT# 104514; RRID: AB_492843
FITC anti-mouse CD25 (Clone PC61)	Biolegend	CAT# 102006; RRID: AB_312855
Pacific Blue anti-mouse/human CD44 (Clone IM7)	Biolegend	CAT# 103020; RRID: AB_493683
PE/Cyanine7 anti-mouse CD62L (Clone MEL-14)	Biolegend	CAT# 104418; RRID: AB_313103
PE anti-mouse CD223 (LAG-3) (Clone C9B7W)	Biolegend	CAT# 125208; RRID: AB_2133343
APC anti-mouse CD366 (Tim-3) (Clone RMT3-23)	Biolegend	CAT# 119706; RRID: AB_2561656
FITC anti-mouse IFN-γ (Clone XMG1.2)	Biolegend	CAT# 505806; RRID: AB_315400
PE anti-human/mouse Granzyme B (Clone QA16A02)	Biolegend	CAT# 372208; RRID: AB_2687032
APC anti-mouse IL-2 (Clone JES6-5H4)	Thermo Fisher Scientific	CAT# 17-7021-82; RRID: AB_469490
PE anti-mouse CD279 (PD-1) (Clone RMP1-30)	Thermo Fisher Scientific	CAT# 12-9981-82; RRID: AB_466290
FITC anti-mouse CD279 (PD-1) (Clone RMP1-30)	Thermo Fisher Scientific	CAT# 11-9981-82; RRID: AB_465467
PerCP anti-human CD45 (Clone HI30)	Biolegend	CAT# 304026; RRID: AB_893337
Pacific Blue anti-human TCR α/β (Clone IP26)	Biolegend	CAT# 306716; RRID: AB_1953257
FITC anti-human CD4 (Clone OKT4)	Biolegend	CAT# 317408; RRID: AB_571951
PE/Cyanine7 anti-human CD4 (Clone OKT4)	Biolegend	CAT# 317414; RRID: AB_571959
APC anti-human CD8 (Clone SK1)	Biolegend	CAT# 344722; RRID: AB_2075388
APC/Cyanine7 anti-human CD8 (Clone SK1)	Biolegend	CAT# 344714; RRID: AB_2044006
PE/Cyanine7 anti-human IFN-γ (Clone B27)	Biolegend	CAT# 506518; RRID: AB_2123321
PE/Cyanine7 anti-human Perforin (Clone dG9)	Biolegend	CAT# 308126; RRID: AB_2572049
APC anti-human TNF-α (Clone MAb11)	Biolegend	CAT# 502912; RRID: AB_315264
APC/Cyanine7 anti-human IL-2 (Clone MQ1-17H12)	Biolegend	CAT# 500341; RRID: AB_2562854
APC anti-human/mouse Granzyme B (Clone QA16A02)	Biolegend	CAT# 372204; RRID: AB_2687028
FITC anti-human CD69 (Clone FN50)	Biolegend	CAT# 310904; RRID: AB_314839
FITC anti-human CD62L (Clone DREG-56)	Biolegend	CAT# 304804; RRID: AB_314464
APC anti-human CD279 (PD-1) (Clone EH12.2H7)	Biolegend	CAT# 329908; RRID: AB_940475
APC/Cyanine7 anti-human CD25 (Clone M-A251)	Biolegend	CAT# 356122; RRID: AB_2562489
PE anti-human TCR Vβ13.1 (Clone H131)	Biolegend	CAT# 362410; RRID: AB_2750159
Human Fc Receptor Blocking Solution (TrueStain FcX)	Biolegend	CAT# 422302; RRID: AB_2818986
Mouse Fc Block (anti-mouse CD16/32)	BD Biosciences	CAT# 553142; RRID: AB_394657
Purified anti-mouse CD3e antibody (Clone 145-2C11)	BD Biosciences	CAT# 553057; RRID: AB_394590
Purified anti-mouse CD28 antibody (Clone 37.51)	BD Biosciences	CAT# 553294; RRID: AB_394763
LEAF purified anti-human CD3 antibody (Clone HIT3a)	Biolegend	CAT# 300314; RRID: AB_314050
LEAF purified anti-human CD28 antibody (Clone CD28.2)	Biolegend	CAT# 302902; RRID: AB_314304
*InVivoMAb* anti-mouse CD8α antibody (Clone 2.43)	BioXCell	CAT# BE0061; RRID: AB_1125541
*InVivo*MAb rat IgG2b isotype control antibody (Clone LTF-2)	BioXCell	CAT# BE0090; RRID: AB_1107780
*InVivo*MAb anti-mouse PD-1 antibody (Clone RMP1-14)	BioXCell	CAT# BE0146; RRID: AB_10949053
*InVivo*MAb rat IgG2a isotype control antibody (Clone 2A3)	BioXCell	CAT# BE0089; RRID: AB_1107769
PE anti-mouse TCF1/TCF7 (clone C63D9)	Cell Signaling Technology	CAT# 14456;RRID: AB_2798483
Anti-mouse NF-κB p65 (Clone D14E12)	Cell Signaling Technology	CAT# 8242S; RRID: AB_10859369
Anti-mouse c-Jun (Clone 60A8)	Cell Signaling Technology	CAT# 9165S; RRID: AB_2130165
Anti-mouse NFAT1	Cell Signaling Technology	CAT# 4389S; RRID: AB_1950418
Anti-mouse Erk1/2 (Clone 3A7)	Cell Signaling Technology	CAT# 9107S; RRID: AB_10695739
Anti-mouse Phospho-Erk1/2 (Clone D13.14.4E)	Cell Signaling Technology	CAT# 4370S; RRID: AB_2315112
Anti-mouse IgG, HRP-linked antibody	Cell Signaling Technology	CAT# 7076S; RRID: AB_330924
Anti-rabbit IgG, HRP-linked antibody	Cell Signaling Technology	CAT# 7074S; RRID: AB_2099233
Anti-GAPDH (Clone 14C10)	Cell Signaling Technology	CAT# 2118S; RRID: AB_561053
Anti-Lamin A/C (Clone 3A6-4C11)	Active Motif	CAT# 39287; RRID: AB_2793218
Bacterial and Virus Strains		
Retro/ESO-TCR	This paper	N/A
Biological Samples		
Human peripheral blood mononuclear cells (PBMCs)	UCLA Center for AIDS Research (CFAR) Virology Core Laboratory	N/A
Chemicals, Peptides, and Recombinant Proteins		
Streptavidin-HRP conjugate	Invitrogen	CAT# 18410051
Mouse IFN-γ (ELISA, standard)	BioLegend	CAT# 575309
Mouse IL-2 (ELISA, standard)	BioLegend	CAT# 575409
Tetramethylbenzidine (TMB)	KPL	CAT# 51200048
Fluoxetine hydrochloride	Abcam	CAT# ab120077
Citalopram	Abcam	CAT# ab120133
Phenelzine sulfate salt	Sigma-Aldrich	CAT# P6777
L-Ascorbic acid	Sigma-Aldrich	CAT# A4403
serotonin receptor (5-HTR) antagonist asenapine	Sigma-Aldrich	CAT# A7861
5-HT2B receptor-selective antagonist RS-127445	Sigma-Aldrich	CAT# R2533
RPMI1640 cell culture medium	Corning Cellgro	CAT# 10-040-CV
DMEM cell culture medium	Corning Cellgro	CAT# 10-013-CV
Fetal Bovine Serum (FBS)	Sigma-Aldrich	CAT# F2442
MACS BSA stock solution	Miltenyi	CAT# 130-091-376
autoMACS Rinsing Solution	Miltenyi	CAT# 130-091-222
Penicillin-Streptomycine-Glutamine (P/S/G)	Gibco	CAT# 10378016
MEM non-essential amino acids (NEAA)	Gibco	CAT# 11140050
HEPES Buffer Solution	Gibco	CAT# 15630056
Sodium Pyruvate	Gibco	CAT# 11360070
β-Mercaptoethanol for cell culture	Sigma-Aldrich	CAT# M3148
Normocin	Invivogen	CAT# ant-nr-2
4′,6-Diamidino-2-Phenylindole, Dilactate (DAPI)	BioLegend	CAT# 422801
Fixable Viability Dye eFluor506	affymetrix eBioscience	CAT# 65-0866-14
Cell Fixation/Permeabilization Kit	BD Biosciences	CAT# 554714
Polybrene infection/transfection reagent	Millipore	CAT# TR-1003-G
10% neutral-buffered formalin	Richard-Allan Scientific	CAT# 5705
Charcoal, Dextran Coated	Sigma-Aldrich	CAT# C6241
Percoll	Sigma-Aldrich	CAT# P4937
Isoflurane	Zoetis	CAT# 50019100
Phosphate Buffered Saline (PBS) pH 7.4 (1X)	Gibco	CAT# 10010-023
Formaldehyde	Sigma-Aldrich	CAT# F8775
Pierce Bovine Serum Albumin Standard	Thermo Fisher Scientific	CAT# 23210
RIPA Lysis and Extraction Buffer	Thermo Fisher Scientific	CAT# 89900
Restore Western Blot Stripping Buffer	Thermo Fisher Scientific	CAT# 21059
Protease/Phosphatase Inhibitor Cocktail	Cell Signaling	CAT# 5872S
4%−15% Mini-PROTEAN^®^ TGX^™^ Precast Protein Gels	Bio-Rad	CAT# 4561084
2-mercaptoethanol	Bio-Rad	CAT# 1610710
4x Laemmli Sample Buffer	Bio-Rad	CAT# 1610747
Trans-Blot Turbo 5x Transfer Buffer	Bio-Rad	CAT# 10026938
10x Tris/Glycine/SDS Buffer	Bio-Rad	CAT# 1610772
Precision Plus Protein Dual Color Standards	Bio-Rad	CAT# 1610394
Blotting Grade Blocker Non Fat Dry Milk	Bio-Rad	CAT# 1706404XTU
Methanol	Thermo Fisher Scientific	CAT# 268280025
Acetonitrile	Thermo Fisher Scientific	CAT# A998SK-1
TRIS-buffered saline (TBS, 10X) pH 7.4	Thermo Fisher Scientific	CAT# J60764.K3
Chloroform	Thermo Fisher Scientific	CAT# C298-1
Ethanol	Thermo Fisher Scientific	CAT# BP2818100
Tween 20	Amresco	CAT# M147-1L
Dimethyl sulfoxide (DMSO)	VWR	CAT# 0231-500ML
TRIzol Reagent	Invitrogen	CAT# 15596018
SsoAdvanced Universal SYBR Green Supermix	Bio-Rad	CAT# 1725271
Golgistop Protein Transport Inhibitor	BD Biosciences	CAT# 554724
Phorbol-12-myristate-13-acetate (PMA)	Sigma-Aldrich	CAT# 524400
Ionomycin calcium salt from Streptomyces conglobatus	Sigma-Aldrich	CAT#I 0634
OVA dextramer	Immudex	CAT# JD2163
Recombinant human IL-2	Peprotech	CAT# 200-02
Critical Commercial Assays		
Serotonin ultrasensitive ELISA kit	Eagle Biosciences	CAT# SEU39-K01
Mouse anti-dsDNA ELISA kit	BioVendor	CAT# 637-02691
Mouse Nave CD8 T Cell Isolation Kit	Miltenyi Biotec	CAT# 130-096-543
Mouse CD8 T Cell Isolation Kit	Miltenyi Biotec	CAT# 130-104-075
Human Naive CD8 T Cell Isolation Kit	Miltenyi Biotec	CAT# 130-093-244
Trans-Blot Turbo RTA Mini 0.2 μm PVDF Transfer Kit	Bio-Rad	CAT# 1704272
Cytiva Amersham ECL Prime Western Blotting Detection Kit	Cytiva	CAT# RPN2232
Fixation/Permeabilization Solution Kit	BD Sciences	CAT# 554714
Foxp3/Transcription Factor Staining Buffer Set	Thermo Fisher Scientific	CAT# 00-5523-00
Bicinchoninic Acid (BCA) Assay Kit	Thermo Fisher Scientific	CAT# 23228 and 1859078
Nuclear Protein Extraction Kit	Thermo Fisher Scientific	CAT# P178833
miRNeasy Mini Kit	Qiagen	CAT# 217004
SuperScript III First-Strand Synthesis Supermix Kit	Invitrogen	CAT# 18080400
Chromium Single Cell 3’ Library & Gel Bead Kit v2	10x Genomics	CAT# PN-120237
NovaSeq 6000 S2 Reagent Kit	Illumina	CAT# 20012862
Deposited Data		
ScRNA-seq of mouse melanoma TIIs(SSRI and anti-PD-1)	Gene expression omnibus	GEO: GSE262781
ScRNA-seq of mouse melanoma TIIs (MAOI)	Gene expression omnibus	GEO: GSE286271
Experimental Models: Cell Lines		
Mouse melanoma cell line B16-OVA	Provided by Dr. Pin Wang (University of Southern California)	N/A
Mouse PG13 cell line	Provided by Dr. Pin Wang (University of Southern California)	N/A
Mouse colon adenocarcinoma cell line MC38	Provided by Dr. Marcus Bosenberg (Yale University)	N/A
Mouse PG13-ESO-TCR stable virus producing cell line	This paper	N/A
Mouse bladder cancer cell line MB49	Provided by Dr. Arnold Qin (University of California, Los Angeles)	N/A
Mouse melanoma cell line B16-F10	ATCC	CAT# CRL-6475
Mouse colon cancer cell line CT26	ATCC	CAT# CRL-2638
Mouse breast cancer cell line 4T1	ATCC	CAT# CRL-2539
Human embryonic kidney 293T cell line	ATCC	CAT# CRL-3216
Human melanoma cell line A375	ATCC	CAT# CRL-1619
Human prostate cancer line PC3	ATCC	CAT# CRL-1435
Human melanoma cell line A375-A2-ESO-FG	This paper	N/A
Human prostate cancer cell line PC3-A2-ESO-FG	This paper	N/A
Experimental Models: Organisms/Strains		
C57BL/6J (B6) mouse	The Jackson Laboratory	Strain #:000664; RRID: IMSR_JAX:000664
BALB/cJ (BALB/c) mouse	The Jackson Laboratory	Strain #:000651; RRID: IMSR_JAX:000651
B6.SJL-*Ptprc*^*a*^*Pepc*^*b*^/BoyJ (CD45.1) mouse	The Jackson Laboratory	Strain #:002014; RRID: IMSR_JAX:002014
C57BL/6-Tg (TcraTcrb)1100Mjb/J (*OT1*-Tg) mouse	The Jackson Laboratory	Strain #:003831; RRID: IMSR_JAX:003831
B6.129(*Cg*)-*Slc6a4*^*tm1Kpl*^*/J* (*Sert*-KO) mouse	The Jackson Laboratory	Strain #:008355; RRID: IMSR_JAX:008355
*B6.129-Htr7*^*tm1Sut*^*/J* (*5Htr7*-KO) mouse	The Jackson Laboratory	Strain #:019453; RRID: IMSR_JAX:019453
*NOD.Cg-Prkdc*^*scid*^ *Il2rg*^*tm1Wjl*^/SzJ (NSG) mouse	The Jackson Laboratory	Strain #:005557; RRID: IMSR_JAX:005557
*OT1*-Tg/*Sert*-KO mouse	This paper	N/A
Recombinant DNA		
Vector: parental pMSGV vector	This paper	N/A
Oligonucleotides		
Primers for quantitative reverse-transcription PCR (RT-qPCR)	Table S1	Table S1
Software and Algorithms		
FlowJo	BD Biosciences	https://www.flowjo.com/solutions/flowjo
Biorender	Biorender	https://www.biorender.com
Photoshop	Adobe	https://www.adobe.com/products/photoshop
I-control 1.7 Microplate Reader Software	Tecan	https://www.selectscience.net/tecan/i-control-microplate-reader-software/81307
ImageJ	NIH	https://imagej.net
Graphpad Prism 9	Graphpad	https://www.graphpad.com/scientific-software/prism
Matlab	MathWorks	http://www.mathworks.com/products/matlab.html
R	R Consortium	http://www.R-project.org
RStudio	RStudio	https://posit.co
ShinyGO 0.77	South Dakota State University	https://bioinformatics.sdstate.edu/go77

## Highlights

- SERT acts as an immune checkpoint restricting CD8 T cell antitumor immunity
- SERT inhibits CD8 T cell antitumor responses by depleting intratumoral serotonin
- SSRIs suppress multiple solid tumors and synergize with anti-PD-1 in mice
- SERT upregulates in TEMRA, and its intratumoral expression links to poor patient survival
