# Supplementary material for: Serotonin transporter inhibits antitumor immunity through regulating the intratumoral serotonin axis
Source: Cell. Author manuscript; Available in PMC 2025 Jul 13. (PMC12255530; doi:10.1016/j.cell.2025.04.032)
Supplement: MMC1 [file NIHMS2084264-supplement-MMC1.pdf]

Cell, Volume *188*

Supplemental information

Serotonin transporter inhibits antitumor immunity through regulating the intratumoral serotonin axis

Bo Li, James Elsten-Brown, Miao Li, Enbo Zhu, Zhe Li, Yuning Chen, Elliot Kang, Feiyang Ma, Jennifer Chiang, Yan-Ruide Li, Yichen Zhu, Jie Huang, Audrey Fung, Quentin Scarborough, Robin Cadd, Jin J. Zhou, Arnold I. Chin, Matteo Pellegrini, and Lili Yang

Supplementary information for

Serotonin transporter inhibits antitumor immunity through regulating the intratumoral serotonin axis

Bo Li,[1,*] James Elsten-Brown,[1] Miao Li,[1] Enbo Zhu,[2] Zhe Li,[1] Yuning Chen,[1] Elliot Kang,[1] Feiyang Ma,[3] Jennifer Chiang,[1] Yan-Ruide Li,[1] Yichen Zhu,[1] Jie Huang,[1] Audrey Fung,[1] Quentin Scarborough,[1] Robin Cadd,[1] Jin J Zhou,[4] Arnold I Chin,[5,6,7] Matteo Pellegrini,[3,8,9] and Lili Yang[1,6,7,10,11,12,13,14,*]

[1]Department of Microbiology, Immunology and Molecular Genetics, University of California, Los Angeles, Los Angeles, CA 90095, USA

[2]Department of Materials Science and Engineering, University of California, Los Angeles, Los Angeles, CA 90095, USA

[3]Department of Molecular, Cell and Developmental Biology, University of California, Los Angeles, Los Angeles, CA 90095, USA

[4]Department of Biostatistics, Fielding School of Public Health, University of California, Los Angeles, Los Angeles, CA, USA

[5]Department of Urology, University of California, Los Angeles, Los Angeles, CA 90095, USA

[6]Eli & Edythe Broad Center of Regenerative Medicine and Stem Cell Research, University of California, Los Angeles, Los Angeles, CA 90095, USA

[7]Jonsson Comprehensive Cancer Center, David Geffen School of Medicine, University of California, Los Angeles, Los Angeles, CA 90095, USA

[8]Bioinformatics Interdepartmental Program, University of California, Los Angeles, Los Angeles, CA 90095, USA

[9]Institute for Quantitative and Computational Biosciences-The Collaboratory, University of California, Los Angeles, Los Angeles, CA 90095, USA

[10]Department of Bioengineering, University of California, Los Angeles, CA 90095, USA

[11]Goodman-Luskin Microbiome Center, University of California, Los Angeles, CA 90095, USA

[12]Molecular Biology Institute, University of California, Los Angeles, Los Angeles, CA 90095, USA

[13]Parker Institute for Cancer Immunotherapy, University of California, Los Angeles, Los Angeles, CA 90095, USA

[14]Lead contact

*Correspondence: bo.li@ucla.edu (B.L.), liliyang@ucla.edu (L.Y.)

The SI includes Supplementary tables 1 and 2, and Supplementary references.

Table S1. Primers used for quantitative reverse-transcription PCR (RT-qPCR), related to STAR Methods

Gene	Forward Primer 5'-3'	Reverse Primer 5'-3'	Source
Ube2d2	ACAAGGAATTGAATGACCTGGC	CACCCTGATAGGGGCTGTC	This paper
Il2	TGAGCAGGATGGAGAATTACAGG	GTCCAAGTTCATCTTCTAGGCAC	This paper
Ifng	ATGAACGCTACACACTGCATC	CCATCCTTTTGCCAGTTCCTC	This paper
Gzmb	CCTCCTGCTACTGCTGAC	GTCAGCACAAAGTCCTCTC	This paper
Tnf	AAGCCTGTAGCCCACGTCGTA	AGGTACAACCCATCGGCTGG	This paper
Perforin	GAGAAGACCTATCAGGACCA	AGCCTGTGGTAAGCATG	This paper
Tph1	CACGAGTGCAAGCCAAGGTTT	AGTTTCCAGCCCCGACATCAG	Wang et al.[S1]
Maoa	CCTGGTATCATGACTCTGTATGG	CTTGGACTCAGGCTCTTGAAC	Wang et al.[S1]
Sert	CGCAGTTCCCAGTACAAGC	CGTGAAGGAGGAGATGAGGT	Chen et al.[S2]
5-Htr1a	ACCCCAACGAGTGCACCATCAG	GCAGGCGGGGACATAGGAG	Wang et al.[S1]
5-Htr1b	CGATGCGGTGGAGTATTCTGC	TAGCGGCCATGAGTTTCTTCTTTT	Wang et al.[S1]
5-Htr1d	CTCTGAGACCCGGGTTGATTT	GGGCCACTGATACCACTTTCCTTA	Wang et al.[S1]
5-Htr1f	CGCAGCGATCACGAGGGAGTT	CGGCGGGCTAGGGACCAGA	Wang et al.[S1]
5-Htr2a	CAGCCCTCCCTCCTCGTTTTG	GCCGGAAGTTGTAGCAGATGAAGT	Wang et al.[S1]
5-Htr2b	AGGATAATTTGGCCCGAGTGCTG	GGTTTCCTTGTCATGCCCGTGT	Wang et al.[S1]
5-Htr2c	TCGTGATTATAATCATGACAATAGGGG	TGAGCACGCAGGTAGTATTATTCACGA	Wang et al.[S1]
5-Htr3	CAACGTGGATGAGAAGAACCAGG	AGCAAGAGGCTGACTGCATAGAATAAAG	Wang et al.[S1]
5-Htr4	CTGGGCTTATGGGGAGATGTTCT	GCTGGGGCCTGCTTTCAGAG	Wang et al.[S1]
5-Htr5a	TGCAGGCGACCAGACAAGAG	CCGACTAGGGCGCAGGACTT	Wang et al.[S1]
5-Htr5b	TCGCCCTGGATCGCTACTGGAC	GCTCGGCGACGGGCTGTGAAC	Wang et al.[S1]
5-Htr6	CTTTGGACCGCCTTCGACGTGATGTGC	GGCTGGCCTTCAAGGCCTTCCTGCTATG	Wang et al.[S1]
5-Htr7	CCGTGAGGCAGAATGGGAAATGTAT	CACTGCGGTGGAGTAGATCGTGTAGC	Wang et al.[S1]
ACTIN	GAGCACAGAGCCTCGCCTTT	ACATGCCGGAGCCGTTGTC	This paper

IL2	AGAACTCAAACCTCTGGAGGAAG	GCTGTCTCATCAGCATATTCACAC	This paper
IFNG	GAGGTGGGAGACCATCAAGGAAG	TGCTTTGCGTTGGACATTCAAGTC	This paper
GZMB	CGACAGTACCATTGAGTTGTGCG	TTCGTCCATAGGAGACAATGCCC	This paper
TNF	CTCTTCTGCCTGCTGCACTTTG	ATGGGCTACAGGCTTGTCACTC	This paper
TPH1	ACGTCGAAAGTATTTTGCGGA	ACGGTTCCCCAGGTCTTAATC	Tow et al.[S3]
MAOA	GTCTTAAATGGTCTCGGGAAGG	CCAGAAGGTGTGGGTGATTT	Wang et al.[S1]
SERT	TGGTTCTATGGCATCACTCAGTTC	GTTGTGGCGGGCTCATCAG	van Lelyveld et al.[S4]
5-HTR1A	GACAGGCGGCAACGATACT	CCAAGGAGCCGATGAGATAGTT	Zhu et al.[S5]
5-HTR1B	CGCCGACGGCTACATTTAC	TAGCTTCCGGGTCCGATACA	Zhu et al.[S5]
5-HTR1D	ATCACCGATGCCCTGGAGTA	GCCAGAAGAGTGGAGGGATG	Zhu et al.[S5]
5-HTR1F	ATCAACTCCCTCGTGATCGC	ACACGTACAACAGATGATGTCG	Zhu et al.[S5]
5-HTR2A	TAATGCAATTAGGTGACGACTCG	GCAGGAGAGGTTGGTTCTGTTT	Zhu et al.[S5]
5-HTR2B	GAACAAAGCACAACTTCTGAGC	CCGCGAGTATCAGGAGAGC	Zhu et al.[S5]
5-HTR2C	CTAATTGGCCTATTGGTTTGGCA	CGGGAATTGAAACAAGCGTCC	Zhu et al.[S5]
5-HTR3A	CCTGGCTAACTACAAGAAGGGG	TGCAGAAACTCATCAGTCCAGTA	Zhu et al.[S5]
5-HTR3B	CTGTCTACCTGGACCTTTGCG	AACTCATCGTTCCAAACCTCTC	Zhu et al.[S5]
5-HTR4	AGTTCCAACGAGGGTTTCAGG	CAGCAGGTTGCCCAAGATG	Zhu et al.[S5]
5-HTR5A	ATGGATCTGCCTGTAAACTTGAC	CACTCGGAAAGCTGAGAGAAAA	Zhu et al.[S5]
5-HTR5B	TTGCTGATCGCTGCCACTTT	GTCGAGGCCACCAAGTTATGT	Zhu et al.[S5]
5-HTR6	GCTGTGCGTGGTCATCGTA	CATCAGGTCCGACGTGAAGAG	Zhu et al.[S5]
5-HTR7	CCGACCTCTACGGCCATCT	TCTCGACTCTGCCATAGTTGAT	Zhu et al.[S5]
FCGR3A	TCGAGCTACTTCATTGACGC	GATATGGACTTCTAGCTGCACC	Morris et al.[S6]
CD300A	TGGGCTCTGTTGCTTCTCTG	GCCGTTCCTTTTTCCTGCTG	Sun et al.[S7]
SYK	TTTCGGACTTTCCAAAGCACTGCG	ACTCCAAAGCTCCAGACATCGCTT	Gao et al.[S8]

ATP7A	GCTACCTTGTCAGACACGAATGAG	TCTTGAACTGGTGTCATCCCTTT	Llanos et al.[S9]
BTBD9	GGCAACGCTGACAGATGAGAA	AGGTAGAATCCTCTAGCTCTGGA	Gao et al.[S8]
CYP2D6	TGATGAGAACCTGCGCATAG	CCCTATCACGTCGTCGATCT	Takemura et al.[S10]
ALDH2	TCAAATTACAGGGTCAACTGCTA	GCCCCCAACAGACCCCAATC	Jin et al.[S11]
XBP1	AAGAACACGCTTGGGAATGG	CTGCACCTGCTGCGGAC	Tan et al.[S12]
GCH1	CGCCTACTCGTCCATCCTGA	CCTTCACAATCACCATCTCA	Tatham et al.[S13]
SLC22A1	TAATGGACCACATCGCTCAA	AGCCCCTGATAGAGCACAGA	Sundelin et al.[S14]
SPR	GACCTGAAAGTGGTGCTGGCAG	GAGGAAGCCTTTGGAAACATCCC	Zhang et al.[S15]

Table S2. Comparison of SERT regulation of 5-HT in neurons and in intratumoral T cells, related to Figure 7

Cell type / Key Differences	Neurons	Intratumoral T cells
**SERT gene expression**	largely constant[S16]	highly dynamic
**SERT-5-HT axis**	paracrine	autocrine & paracrine
**5-HT receptor usage & signaling pathways**	5-HT1A, 5-HT2A, 5-HT2C[S17] & PLC/IP3/DAG/PKC, cAMP/PKA signaling pathways[S18]	5-HT2B, 5-HT7, 5-HT4 & MAPK and TCR signaling pathways

SUPPLEMENTAL REFERENCES

S1. Wang, X., Li, B., Kim, Y.J., Wang, Y.C., Li, Z., Yu, J., Zeng, S., Ma, X., Choi, I.Y., Di Biase, S., et al. (2021). Targeting monoamine oxidase A for T cell-based cancer immunotherapy. Sci Immunol *6*. https://doi.org/10.1126/sciimmunol.abh2383.

S2. Chen, X., Margolis, K.J., Gershon, M.D., Schwartz, G.J., and Sze, J.Y. (2012). Reduced serotonin reuptake transporter (SERT) function causes insulin resistance and hepatic steatosis independent of food intake. PLoS One *7*, e32511. https://doi.org/10.1371/journal.pone.0032511.

S3. Tow, D.H., Tran, C.G., Borbon, L.C., Ridder, M., Li, G., Kaemmer, C.A., Abusada, E., Mahalingam, A.H., Sadanandam, A., Chandrasekaran, C., et al. (2023). Inhibition of serotonin biosynthesis in neuroendocrine neoplasm suppresses tumor growth in vivo. bioRxiv. https://doi.org/10.1101/2023.04.07.536013.

S4. van Lelyveld, N., Ter Linde, J., Schipper, M.E.I., and Samsom, M. (2007). Regional differences in expression of TPH-1, SERT, 5-HT(3) and 5-HT(4) receptors in the human stomach and duodenum. Neurogastroenterology and motility *19*, 342–348. https://doi.org/10.1111/j.1365-2982.2006.00891.x.

S5. Zhu, P., Lu, T., Chen, Z., Liu, B., Fan, D., Li, C., Wu, J., He, L., Zhu, X., Du, Y., et al. (2022). 5-hydroxytryptamine produced by enteric serotonergic neurons initiates colorectal cancer stem cell self-renewal and tumorigenesis. Neuron *110*, 2268-2282.e4. https://doi.org/10.1016/j.neuron.2022.04.024.

S6. Morris, M.C., Nadeem Khan, M., and Pichichero, M.E. (2017). A PCR-based method for quantifying neutrophils in human nasal secretions. J Immunol Methods *447*, 65–70. https://doi.org/10.1016/j.jim.2017.04.010.

S7. Sun, X., Huang, S., Wang, X., Zhang, X., and Wang, X. (2018). CD300A promotes tumor progression by PECAM1, ADCY7 and AKT pathway in acute myeloid leukemia. Oncotarget *9*, 27574–27584. https://doi.org/10.18632/oncotarget.24164.

S8. Gao, D., Wang, L., Zhang, H., Yan, X., Yang, J., Zhou, R., Chang, X., Sun, Y., Tian, S., Yao, Z., et al. (2018). Spleen tyrosine kinase SYK(L) interacts with YY1 and coordinately suppresses SNAI2 transcription in lung cancer cells. FEBS J *285*, 4229–4245. https://doi.org/10.1111/febs.14665.

S9. Llanos, R.M., Ke, B.-X., Wright, M., Deal, Y., Monty, F., Kramer, D.R., and Mercer, J.F.B. (2006). Correction of a mouse model of Menkes disease by the human Menkes gene. Biochim Biophys Acta *1762*, 485–493. https://doi.org/10.1016/j.bbadis.2005.12.011.

S10. Takemura, A., Gong, S., Sato, T., Kawaguchi, M., Sekine, S., Kazuki, Y., Horie, T., and Ito, K. (2021). Evaluation of Parent- and Metabolite-Induced Mitochondrial Toxicities Using CYP-Introduced HepG2 cells. J Pharm Sci *110*, 3306–3312. https://doi.org/10.1016/j.xphs.2021.06.001.

S11. Jin, S., Chen, J., Chen, L., Histen, G., Lin, Z., Gross, S., Hixon, J., Chen, Y., Kung, C., Chen, Y., et al. (2015). ALDH2(E487K) mutation increases protein turnover and promotes murine hepatocarcinogenesis. Proc Natl Acad Sci U S A *112*, 9088–9093. https://doi.org/10.1073/pnas.1510757112.

S12. Tan, Z., Zhang, W., Sun, J., Fu, Z., Ke, X., Zheng, C., Zhang, Y., Li, P., Liu, Y., Hu, Q., et al. (2018). ZIKV infection activates the IRE1-XBP1 and ATF6 pathways of unfolded protein response in neural cells. J Neuroinflammation *15*, 275. https://doi.org/10.1186/s12974-018-1311-5.

S13. Tatham, A.L., Crabtree, M.J., Warrick, N., Cai, S., Alp, N.J., and Channon, K.M. (2009). GTP cyclohydrolase I expression, protein, and activity determine intracellular tetrahydrobiopterin levels, independent of GTP cyclohydrolase feedback regulatory protein expression. J Biol Chem *284*, 13660–13668. https://doi.org/10.1074/jbc.M807959200.

S14. Sundelin, E.I.O., Gormsen, L.C., Heebøll, S., Vendelbo, M.H., Jakobsen, S., Munk, O.L., Feddersen, S., Brøsen, K., Hamilton-Dutoit, S.J., Pedersen, S.B., et al. (2019). Hepatic exposure of metformin in patients with non-alcoholic fatty liver disease. Br J Clin Pharmacol *85*, 1761–1770. https://doi.org/10.1111/bcp.13962.

S15. Zhang, X., Chen, Y., Wang, K., Tang, J., Chen, Y., Jin, G., and Liu, X. (2020). The knockdown of the sepiapterin reductase gene suppresses the proliferation of breast cancer by inducing ROS-mediated apoptosis. Int J Clin Exp Pathol *13*, 2228–2239.

S16. Blakely, R.D., and Edwards, R.H. (2012). Vesicular and Plasma Membrane Transporters for Neurotransmitters. Cold Spring Harb Perspect Biol *4*, a005595–a005595. https://doi.org/10.1101/cshperspect.a005595.

S17. Albert, P.R., Le François, B., and Millar, A.M. (2011). Transcriptional dysregulation of 5-HT1A autoreceptors in mental illness. Mol Brain *4*, 21. https://doi.org/10.1186/1756-6606-4-21.

S18. Bubak, A.N., Watt, M.J., Yaeger, J.D.W., Renner, K.J., and Swallow, J.G. (2020). The stalk-eyed fly as a model for aggression – is there a conserved role for 5-HT between vertebrates and invertebrates? Journal of Experimental Biology *223*. https://doi.org/10.1242/jeb.132159.
