# Supplementary material for: Serotonin transporter inhibits antitumor immunity through regulating the intratumoral serotonin axis
Source: Cell. Author manuscript; Available in PMC 2025 Jul 13. (PMC12255530; doi:10.1016/j.cell.2025.04.032)
Supplement: 1 [file NIHMS2084264-supplement-1.pdf]

CellPress
OPEN ACCESS

Supplemental figures

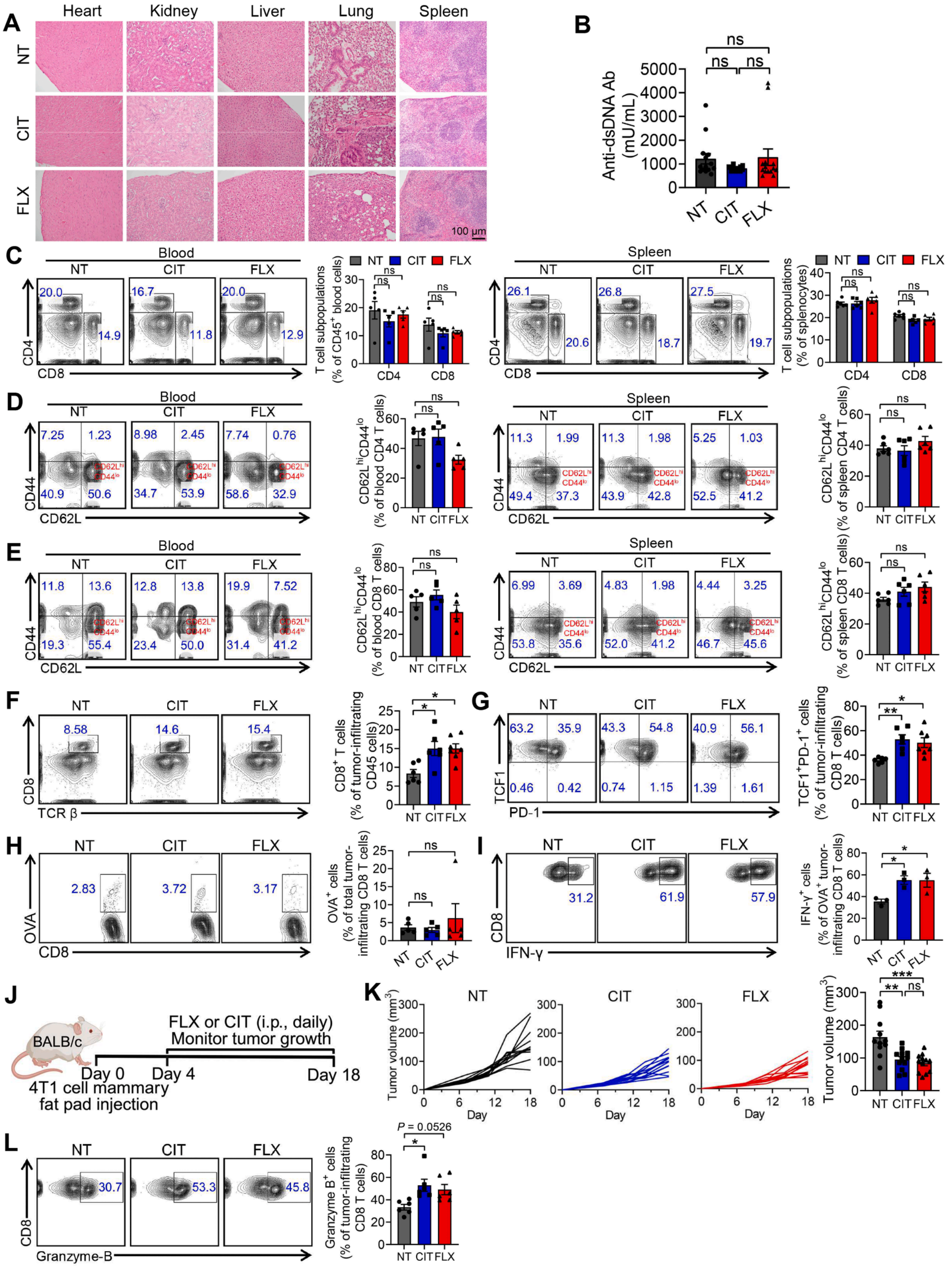

(legend on next page)

Figure S1. SERT blockade is generally safe and enhances CD8 T cell antitumor immunity in syngeneic mouse tumor models, related to Figure 1

(A–I) SSRI treatment in a B16-OVA syngeneic mouse melanoma model, related to Figures 1E–1H. Experimental design is shown in Figure 1E. Experimental animals were analyzed at day 14. (A) H&E-stained tissue sections ($n = 5$). Scale bar: 100 μm. (B) ELISA analyses of serum autoantibodies against dsDNA ($n = 8$–10). Ab, antibody; dsDNA, double-stranded DNA. (C) FACS quantification of CD4 and CD8 T cells (gated as $CD45^{+}CD4^{+}$ and $CD45^{+}CD8^{+}$ cells, respectively) in the peripheral blood and spleen ($n = 5$). (D and E) FACS analyses of surface activation markers (i.e., CD44 and CD62L) on CD4 T cells (pre-gated as $CD45^{+}CD4^{+}$ cells; D) and CD8 T cells (pre-gated as $CD45^{+}CD8^{+}$ cells; E) in the peripheral blood and spleen ($n = 6$). (F and G) FACS quantification of CD8 T cells (gated as $CD45^{+}TCR\beta^{+}CD8^{+}$ cells; F) and their "stem-like" memory subset (gated as $CD45^{+}TCR\beta^{+}CD8^{+}PD\text{-}1^{+}TCF1^{+}$ cells; G) in B16-OVA tumors ($n = 6$–7). (H and I) FACS quantification of OVA-specific CD8 T cells (gated as $CD45^{+}TCR\beta^{+}CD8^{+}OVA\text{-}tetramer^{+}$ cells; H) and their intracellular IFN-γ production (I) in B16-OVA tumors ($n = 4$–5).

(J–L) SSRI treatment in a 4T1 syngeneic mouse breast cancer orthotopic model. (J) Experimental design. (K) Tumor growth ($n = 11$–12). (L) FACS analyses of intracellular Granzyme B production in tumor-infiltrating CD8 T cells isolated from day 18 4T1 tumors ($n = 6$).

Representative of one (A), two (B, F–I, K, and L), and three (C–E) experiments. Data are presented as the mean ± SEM. ns, not significant, $^{*}p < 0.05$, $^{**}p < 0.01$, and $^{***}p < 0.001$ by one-way ANOVA (B–I, K, and L).

CellPress
OPEN ACCESS

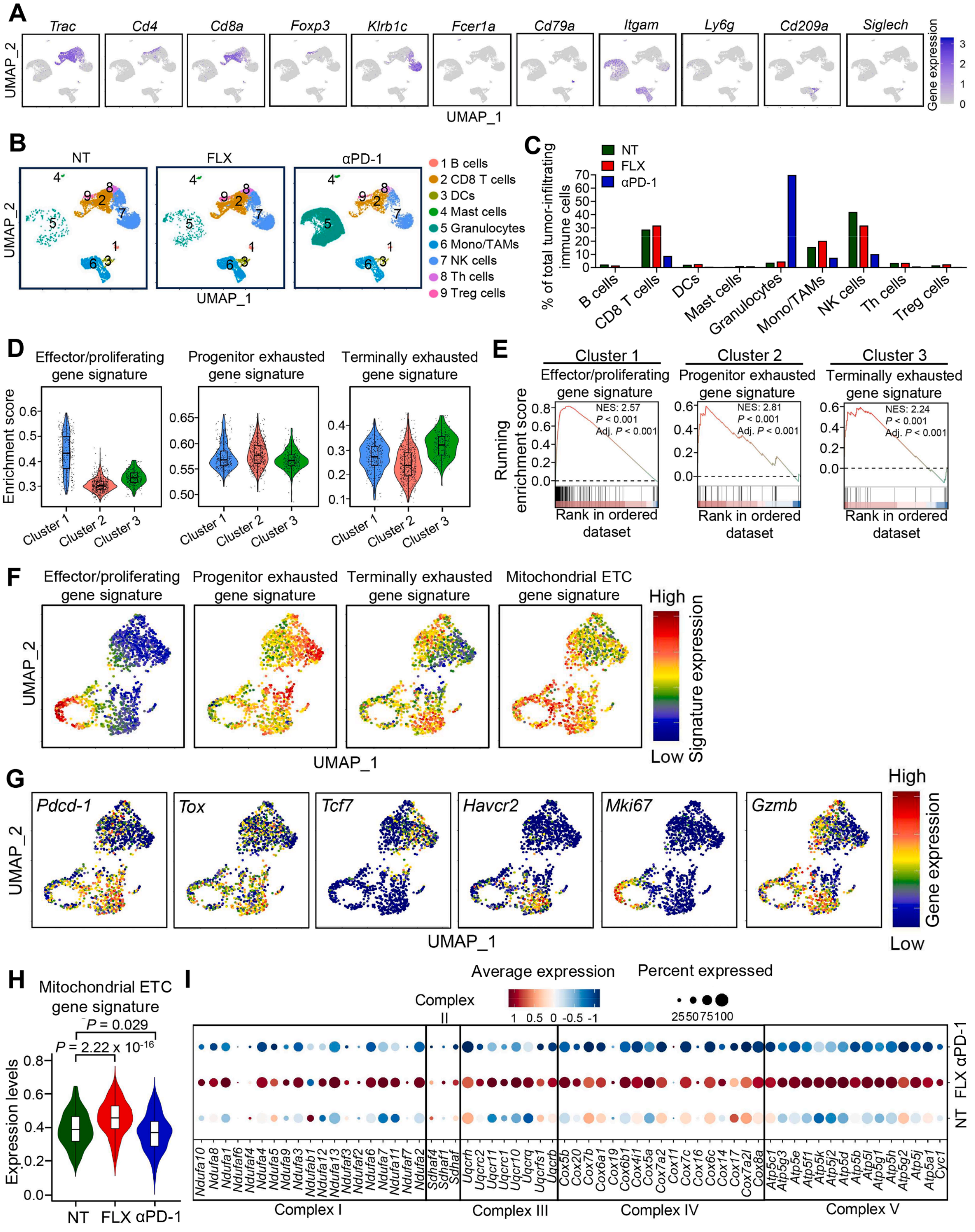

(legend on next page)

Figure S2. SERT blockade enhances antitumor CD8 T cell effector and proliferating gene profiles, related to Figure 2

(A–C) Profiling the total CD45$^+$ TIIs, related to Figure 2B. (A) Combined UMAP plots showing the expression patterns of 11 marker genes used to define the 9-cell clusters. Each dot represents one single cell and is colored according to its expression of the indicated marker gene. (B) Individual UMAP plots showing the nine-cell cluster composition of the indicated experimental groups. (C) Quantification of (B).

(D–I) Profiling the antigen-experienced (CD44$^+$) tumor-infiltrating CD8 T cells, related to Figure 2C. (D) Violin plots showing the expression distribution of the indicated gene signatures in the three-cell clusters, validating their definition of cluster 1 to be the effector/proliferating CD8 T cells, cluster 2 to be the progenitor exhausted CD8 T cells, and cluster 3 to be the terminally exhausted CD8 T cells. (E) Gene set enrichment analysis (GSEA) plots showing the enrichment of the indicated gene signatures in cluster 1, 2, and 3 cells. (F) UMAP plots showing the expression of the indicated gene signatures. Each dot represents one single cell and is colored according to its expression of the indicated gene signature. ETC, electron transport chain. (G) UMAP plots showing the expression of the indicated marker genes. Each dot represents one single cell and is colored according to its expression of the indicated marker gene. (H) Violin plots showing the expression distribution of the mitochondrial ETC gene signature in the indicated treatment groups. A total of 80 genes encoding the ETC complexes I–V were studied. Box and whisker plots exhibit the minimum, lower quartile, median, upper quartile, and maximum expression levels of each group. (I) Dot plots displaying the expression of individual mitochondrial ETC genes in the indicated treatment groups. Color saturation indicates the strength of averaged gene expression. The dot size indicates the percentage of cells expressing the indicated genes.

The experiment was performed once, and cells isolated from 10 mice of each experimental group were combined for analysis. The p values of violin plots were determined by the Kruskal-Wallis test for the overall comparison and Dunn's test for post hoc pairwise comparisons between groups (H). $p < 0.05$ was considered significant.

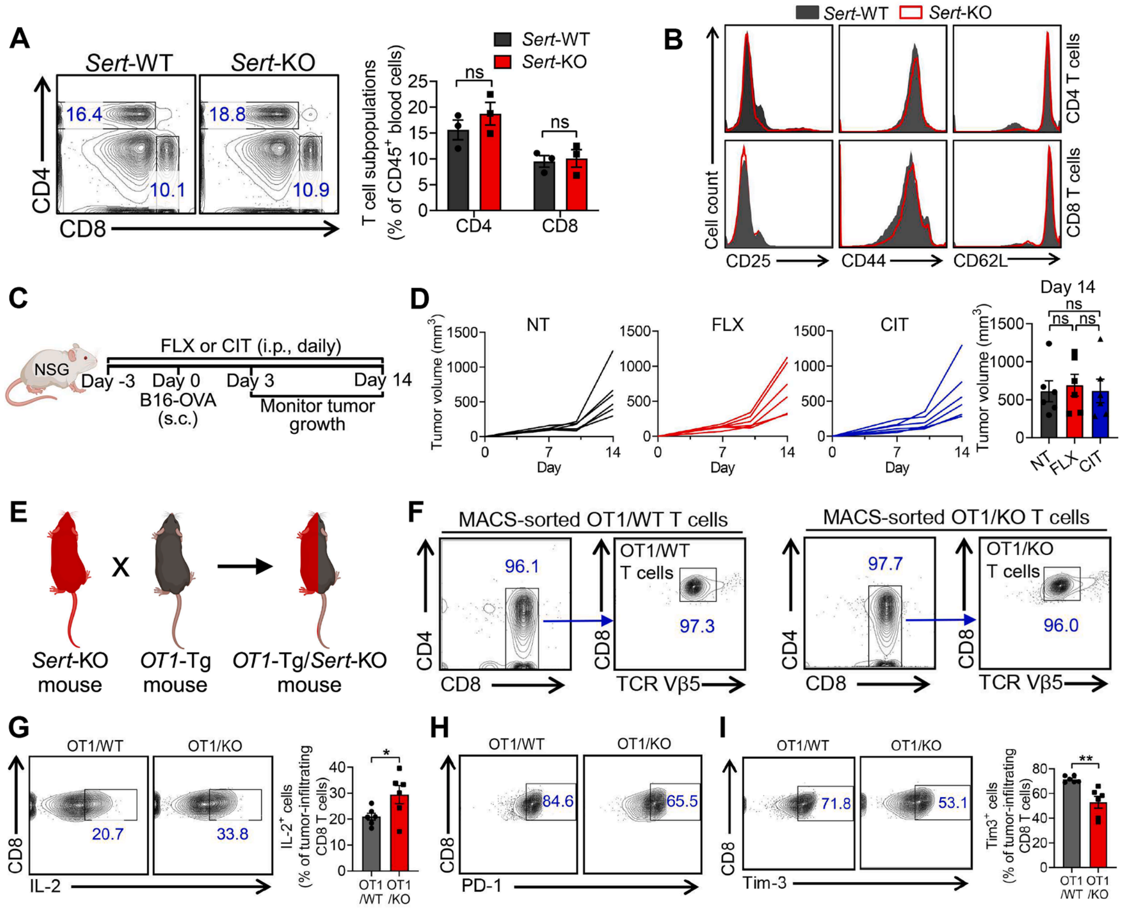

Figure S3. SERT functions as a T cell-intrinsic factor negatively regulating CD8 T cell-mediated antitumor responses, related to Figure 3

(A and B) Comparing the T cell compartment in *Sert*-KO and *Sert*-WT mice (n = 3). FACS analyses of blood cells are presented, showing the detection of CD4 and CD8 T cells (identified as CD45$^+$CD4$^+$ and CD45$^+$CD8$^+$ cells, respectively) in comparable numbers (A) and of a similar CD25^{lo}CD44^{lo}CD62L^{hi} naive T cell phenotype (B).

(C and D) NSG immunodeficient mice tumor challenge experiment. (C) Experimental design. The B16-OVA mouse melanoma model and two SSRIs (FLX and CIT) were used. (D) Tumor growth (n = 6). NT, non-treated.

(E) Breeding strategy to generate the OT1 transgenic (*OT1*-Tg) mice deficient of *Sert* gene (denoted as the *OT1*-Tg/*Sert*-KO mice).

(F–I) OT1 T cell adoptive transfer experiment. Experimental design is shown in Figure 3L. (F) FACS analyses of OT1 transgenic T cells sorted from the spleens of *OT1*-Tg and *OT1*-Tg/*Sert*-KO mice (>95% purity, gated as CD4$^-$CD8$^+$TCR Vβ5$^+$ cells; denoted as the OT1/WT and OT1/KO T cells, respectively). (G–I) FACS analyses of intracellular IL-2 production (G) and surface PD-1 (H) and Tim-3 (I) expression in tumor-infiltrating CD8 T cells isolated from day 17 B16-OVA tumors (n = 6).

Representative of one (F–I) and two (A, B, and D) experiments. Data are presented as the mean ± SEM. ns, not significant, *p < 0.05 and **p < 0.01, by Student's t test (A, G, and I) or one-way ANOVA (D).

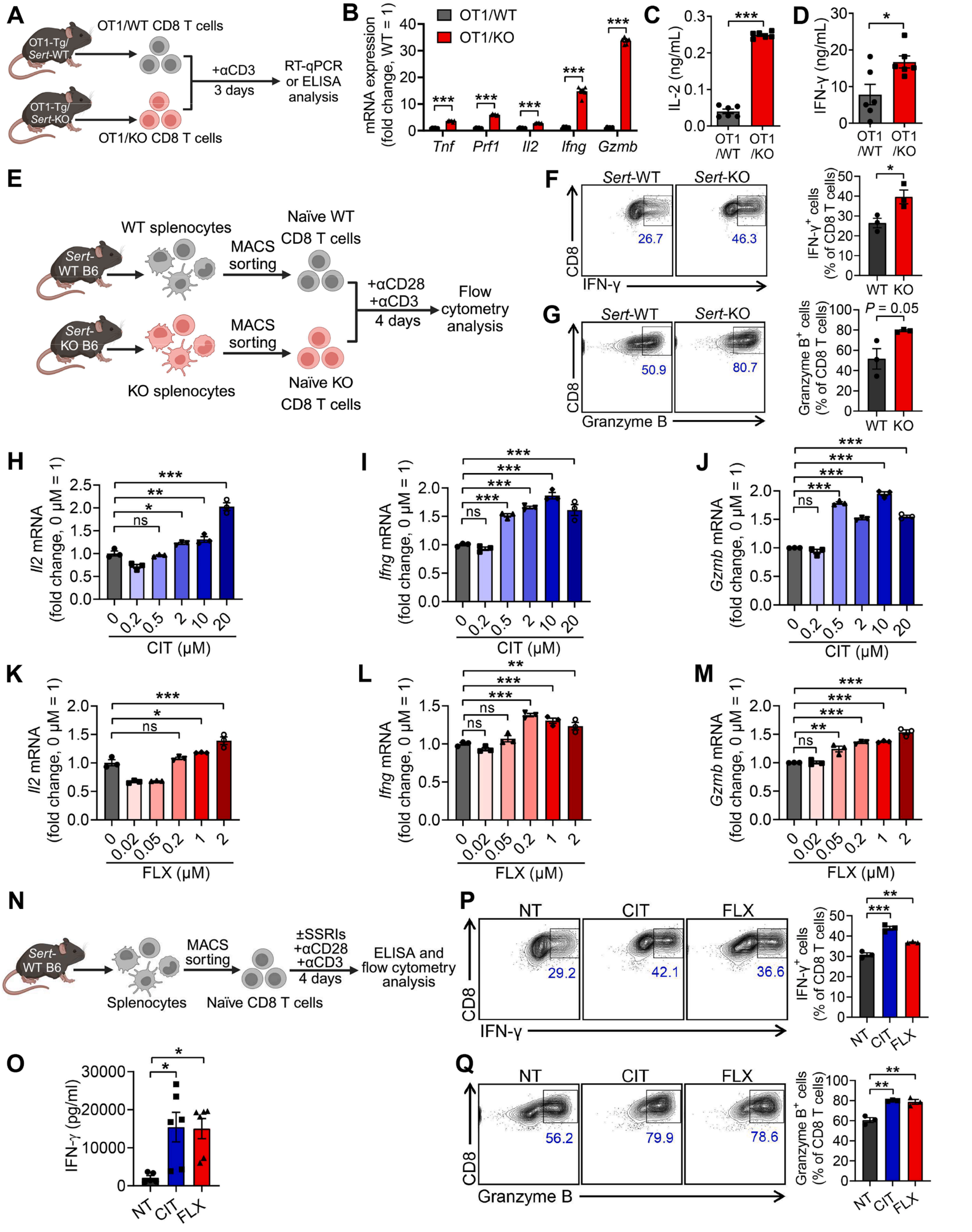

(legend on next page)

Figure S4. SERT acts as an autonomous factor negatively regulating CD8 T cell antigen responses, related to Figure 4

(A–D) Antigen response of OVA-specific OT1 transgenic CD8 T cells in the absence of SERT. (A) Experimental design. OT1 transgenic CD8 T cells were isolated from the OT1/WT or OT1/KO mice and then stimulated *in vitro* with anti-CD3. (B) RT-qPCR analyses of T cell effector genes at day 2 (n = 6). (C and D) ELISA analyses of IL-2 (C) and IFN-γ (D) production at day 3 (n = 6).

(E–G) CD8 T cell antigen response to anti-CD3/anti-CD28 stimulation in the absence of SERT. (E) Experimental design. CD8 T cells were purified from *Sert*-WT and *Sert*-KO mice and stimulated *in vitro* with anti-CD3 and anti-CD28 over 4 days. (F and G) FACS analyses of intracellular IFN-γ (F) and Granzyme B (G) production at day 3 (n = 3).

(H–M) CD8 T cell antigen response under varying doses of SSRI treatment. Experimental design is shown in Figure 4H. (H–J) RT-qPCR analyses of *Il2* (H), *Ifng* (I), and *Gzmb* (J) expression in CIT-treated CD8 T cells at day 2 (n = 3). (K–M) RT-qPCR analyses of *Il2* (K), *Ifng* (L), and *Gzmb* (M) expression in FLX-treated CD8 T cells at day 2 (n = 3).

(N–Q) CD8 T cell antigen response to anti-CD3/anti-CD28 stimulation under SSRI treatment. (N) Experimental design. CD8 T cells were purified from *Sert*-WT mice and stimulated *in vitro* with anti-CD3 and anti-CD28 in the presence or absence of SSRI (CIT or FLX) treatment for 4 days. NT, non-treated. (O) ELISA analyses of IFN-γ levels in the cell culture supernatants at day 4 (n = 6). (P and Q) FACS analyses of intracellular IFN-γ (P) and Granzyme B (Q) production at day 3 (n = 3).

Representative of one (B–D) and two (F–M and O–Q) experiments. Data are presented as the mean ± SEM. ns, not significant, *$p < 0.05$, **$p < 0.01$, and ***$p < 0.001$ by Student's t test (B–D, F, and G) or one-way ANOVA (H–M and O–Q).

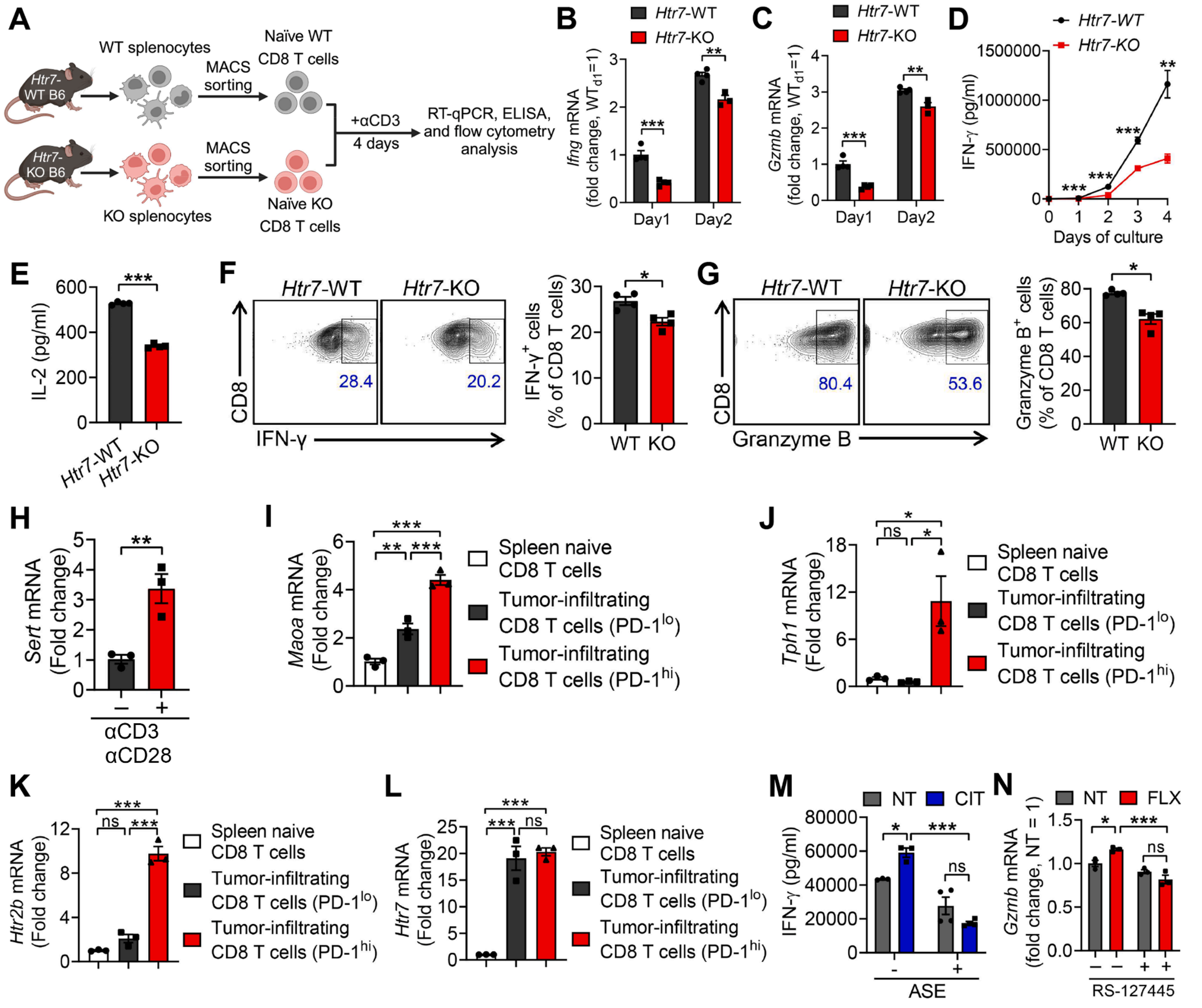

Figure S5. SERT restrains CD8 T cell antigen responses by directly regulating the autocrine serotonin signaling pathway, related to Figure 5

(A–G) CD8 T cell antigen response in the absence of 5-HT7 receptor. (A) Experimental design. CD8 T cells were purified from *Htr7*-WT and *Htr7*-KO mice and stimulated *in vitro* with anti-CD3 over 4 days. (B and C) RT-qPCR analyses of *Ifng* (B) and *Gzmb* (C) gene expression at day 1 and day 2 (n = 3–4). (D) ELISA analyses of IFN-γ production over 4 days (n = 4). (E) ELISA analyses of IL-2 production at day 3 (n = 4). (F and G) FACS analyses of intracellular IFN-γ (F) and Granzyme B (G) production at day 3 (n = 4).

(H) RT-qPCR analyses of *Sert* gene expression in naive CD8 T cells prior to and after TCR stimulation (n = 3). Naive CD8 T cells were isolated from WT B6 mice and simulated with anti-CD3 and anti-CD28 for 3 days.

(I–L) RT-qPCR analyses of *Maoa* (I), *Tph1* (J), *Htr2b* (K), and *Htr7* (L) expression in tumor-infiltrating CD8 T cell subsets (gated as CD45.2$^+$TCRβ$^+$CD8$^+$PD-1^{lo} or CD45.2$^+$TCRβ$^+$CD8$^+$PD-1^{hi}) isolated from day 14 B16-OVA tumors grown in WT B6 mice (n = 3). Naive CD8 T cells (gated as TCRβ$^+$CD8$^+$CD44^{lo}CD62L^{hi}) sorted from the spleens of tumor-free WT B6 mice were included as a control.

(M and N) Autocrine serotonin signaling in *Sert*-WT CD8 T cells. Experimental design is shown in Figure 5I. (M) ELISA analyses of IFN-γ levels in CIT-treated CD8 T cell culture supernatants at day 3, with or without ASE treatment (n = 3–4). (N) RT-qPCR analyses of *Gzmb* mRNA expression in FLX-treated or non-treated (NT) CD8 T cells at day 2, with or without RS-127445 treatment (n = 3).

Representative of two experiments (B–N). Data are presented as the mean ± SEM. ns, not significant, *p < 0.05, **p < 0.01, and ***p < 0.001 by Student's t test (B–H), one-way ANOVA (I–L), or two-way ANOVA (M and N).

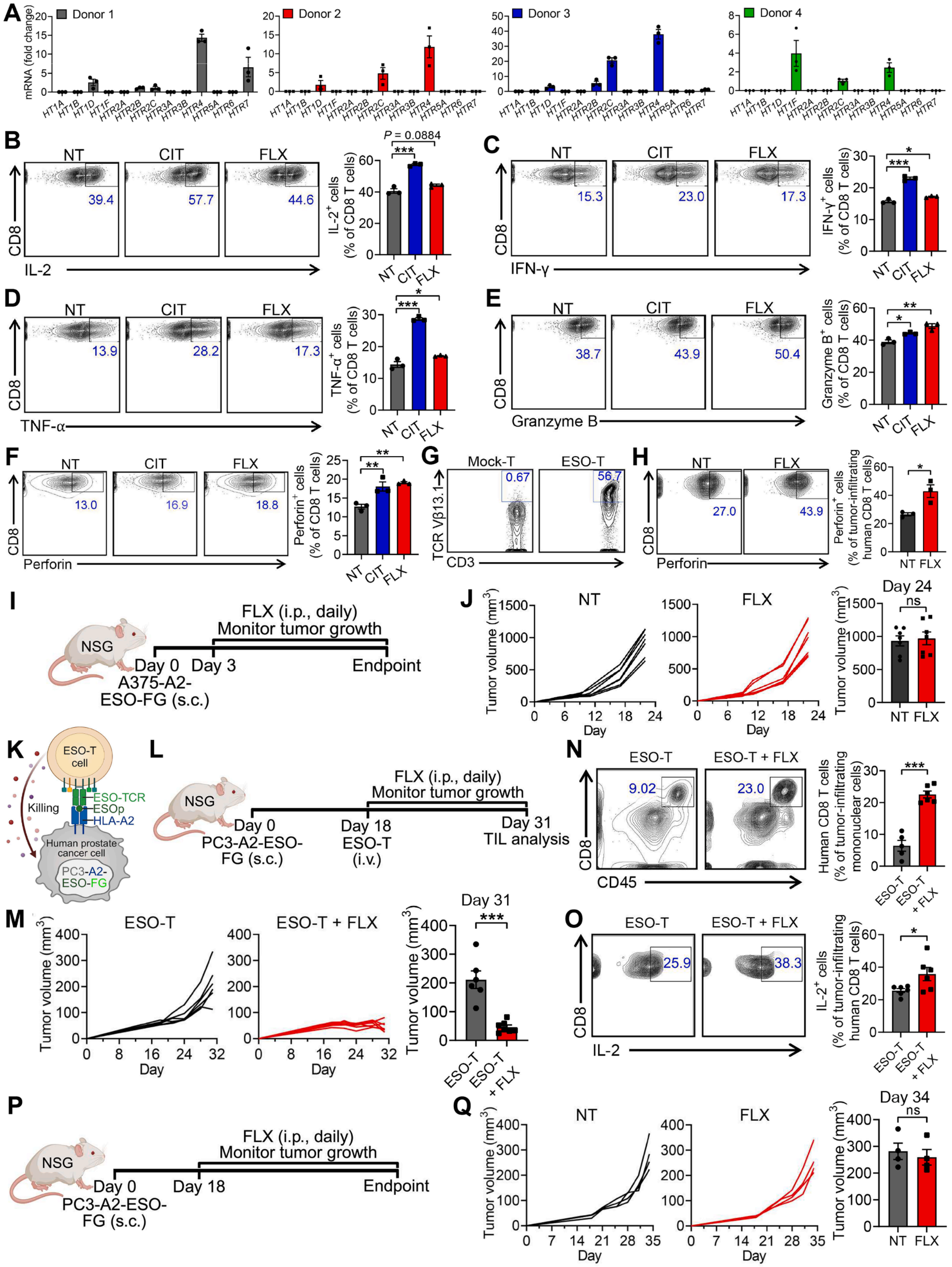

(legend on next page)

Figure S6. SERT blockade for cancer immunotherapy: Human T cell studies, related to Figure 6

(A–F) Studying human CD8 T cell antigen responses under SSRI treatment. Experimental design is shown in Figure 6A. Human naive CD8 T cells were sorted from healthy donor PBMCs and stimulated with anti-CD3/anti-CD28/IL-2 *in vitro* for 5 days in the absence (non-treated, NT) or presence of SSRI (FLX or CIT) treatment. (A) RT-qPCR analyses of 5-HTR family member gene expression in stimulated human CD8 T cells from 4 different healthy donors ($n = 3$ per donor). (B–F) FACS analyses of intracellular IL-2 (B), IFN-γ (C), TNF-α (D), Granzyme B (E), and Perforin (F) production at day 5 ($n = 3$).

(G) FACS analyses of ESO-TCR expression on the engineered ESO-T cells. ESO-T cell design is depicted in Figure 6K. Human CD8 T cells that received mock transduction were included as a control (denoted as Mock-T cells).

(H) FACS analyses of intracellular Perforin production in tumor-infiltrating CD8 T cells isolated from day 15 A375-A2-ESO-FG tumors without (non-treated, NT) or with FLX treatment ($n = 3$). Experimental design is shown in Figure 6L.

(I and J) Studying the A375-A2-ESO-FG human melanoma growth in NSG mice without (non-treated, NT) or with FLX treatment. (I) Experimental design. (J) Tumor growth ($n = 7$).

(K–Q) Studying SERT blockade in a serotonin-secreting PC3 human prostate neuroendocrine cancer xenograft model. (K) Schematics showing a human tumor cell-T cell pair designated for this study. PC3-A2-ESO-FG: PC3 human prostate cancer cell line engineered to co-express the tumor antigen NY-ESO-1, its matching MHC molecule (HLA-A2), as well as the firefly luciferase and enhanced green fluorescence protein dual-reporters (FG); ESO-T: human healthy donor peripheral blood CD8 T cells engineered to express an NY-ESO-1-specific TCR. (L–O) Studying SSRI (i.e., FLX) treatment in combination with ESO-T cell adoptive transfer. (L) Experimental design. (M) Tumor growth ($n = 6–7$). (N and O) FACS analyses of ESO-T cell abundance (N) and intracellular IL-2 production (O) in day 31 tumors ($n = 5–6$).

(P and Q) Studying SSRI (i.e., FLX) treatment without ESO-T cell adoptive transfer. (P) Experimental design. (Q) Tumor growth ($n = 4$).

Representative of one (A) and two (B–H, J, M–O, and Q) experiments. Data are presented as the mean ± SEM. ns, not significant, $^{*}p < 0.05$, $^{**}p < 0.01$, and $^{***}p < 0.001$ by one-way ANOVA (B–F) or Student's t test (H, J, M–O, and Q).

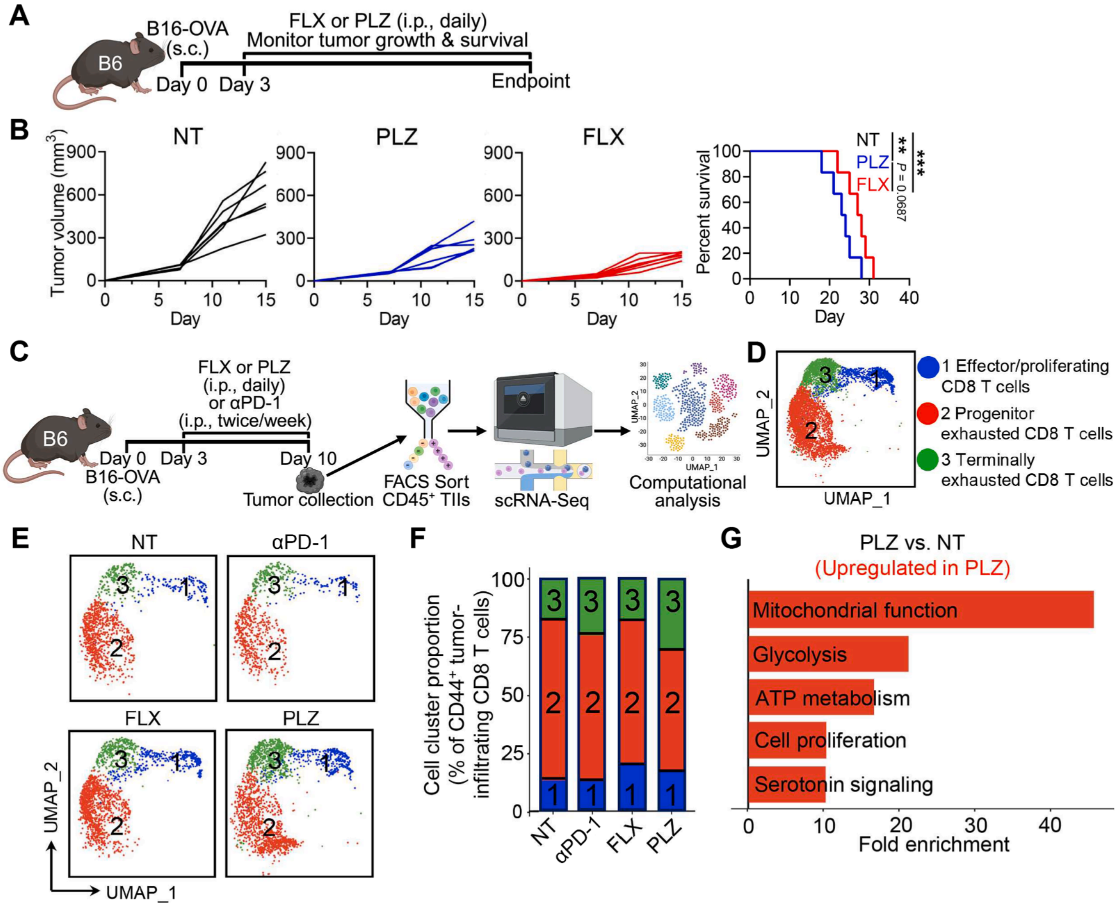

Figure S7. Comparison of SSRI and MAOI treatments in a B16-OVA melanoma model, related to Figures 1 and 2

(A and B) *In vivo* antitumor efficacy study. MAOI, monoamine oxidase inhibitor. FLX (fluoxetine; an SSRI) and phenelzine (PLZ) (an MAOI) were used in the study. (A) Experimental design. (B) Tumor growth and survival of the non-treated (NT), PLZ-treated (PLZ), and FLX-treated (FLX) experimental animals ($n = 6$).

(C–G) scRNA-seq study. (C) Experimental design. $CD45^+$ tumor-infiltrating immune cells (TIIs) were sorted from day 10 B16-OVA tumors and then subjected to scRNA-seq analysis. Four experimental groups were included: non-treated (NT), anti-PD-1-treated (αPD-1), FLX-treated (FLX), and PLZ-treated (PLZ). 10 tumors were combined from each experimental group for analysis. (D) Combined UMAP plot showing the formation of three major cell clusters of antigen-experienced ($CD44^+$) tumor-infiltrating CD8 T cells. Total cells combined from all samples are included. Each dot represents a single cell and is colored according to its cell cluster assignment. (E) Individual UMAP plots showing cell cluster composition of the indicated treatment groups. (F) Bar graphs showing the cell cluster proportions of the indicated treatment groups. (G) Bar graphs showing the fold enrichment of indicated pathways upregulated under the PLZ treatment. Representative of one (D–G) and three (B) experiments. $^{**}p < 0.01$ and $^{***}p < 0.001$, by log rank (Mantel-Cox) test (B).
